# Should the age range of the Dutch hrHPV‐based cervical cancer screening program be broadened? A modelling study using cohort effects

**DOI:** 10.1002/ijc.35435

**Published:** 2025-04-28

**Authors:** Sylvia Kaljouw, Erik E. L. Jansen, Veerle J. C. Schevenhoven, Inge M. C. M. de Kok

**Affiliations:** ^1^ Department of Public Health, Erasmus MC University Medical Center Rotterdam Rotterdam the Netherlands

**Keywords:** age range, cohort effects, HPV vaccination, hrHPV‐based screening, simulation model

## Abstract

In the Netherlands, women are invited for human papillomavirus (HPV) screening between the ages of 30 and 60 (with conditional screening at age 65). However, an increase in cervical cancer (CC) incidence has been observed in younger women recently. Meanwhile, HPV‐vaccinated cohorts reached the screening age of 30 in 2023. Moreover, increasing healthy life expectancy is a consideration for screening in older age groups. Due to these developments, the starting and ending ages of the HPV screening programs should be reconsidered. Microsimulation model MISCAN‐Cervix was recalibrated for cohort effects using updated CC incidence data. We used this model to calculate the cost‐effectiveness of screening unvaccinated women in birth cohorts 1962–1992 until 65 years old. Additionally, we considered starting screening at 25 for partly vaccinated cohorts (born in 2002–2006). Vaccination effects were calculated using microsimulation model STDSIM. Main outcome measures included cancers prevented, life years gained (LYG), costs, and referrals compared to the current strategy (2027 onwards). Adding screening at age 65 to the current strategy leads to +3.5% cancers prevented, +10.3% referrals, +2.4% LYG and +57.0% costs (cost‐effectiveness ratio: €275,096/LYG). Adding screening at age 25 results in extra cases prevented (+1.3%–5.7%, depending on the target group's vaccination status) and LYG (+0.8%–3.7%), but increases referrals (12.9%–37.1%) and costs (+14.0%–33.1%) (cost‐effectiveness ratio: €120,017–€323,813/LYG). So, screening unvaccinated women at 65 years old and screening women in (partly‐)vaccinated cohorts at age 25 might not represent good value for money.

## Graphical abstract

What's new?

Cervical cancer incidence in the Netherlands is increasing among women in younger birth cohorts. This raises concerns about the start and end ages of the country's human papillomavirus (HPV) screening program, which currently includes women ages 30 to 60. Here, the authors estimated the impact of a broader HPV screening age range on cancer prevention and costs in the Netherlands. Models show that expanding screening to cover women ages 25 to 65 modestly benefits cancer prevention and significantly increases unnecessary referrals. The findings indicate that expanding the HPV screening age range to include younger and older women is not cost‐effective.

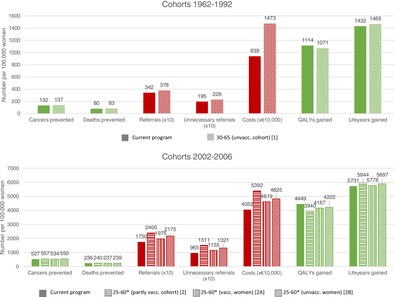

**Abbreviations**
ASC‐US: atypical squamous cells of undetermined significance
CC: cervical cancer
CER: cost‐effectiveness ratio
CIN: cervical intraepithelial neoplasia
FIGO: International Federation of Gynecology and Obstetrics
HPV: Human papillomavirus
Hr: high risk
HSIL: high‐grade squamous intraepithelial lesion
LYG: Lifeyears gained
NNR: Number needed to refer
NNS: Number needed to screen
OHR: other high risk
PPV: positive predictive value
QALY: quality adjusted lifeyear

## 1 INTRODUCTION

In the Netherlands, women are invited for human papillomavirus (HPV) screening every 5 years between the ages of 30 and 60. In this program, women are only screened at age 45 and/or 55 if they did not participate or got a positive HPV test result (without referral) in the previous round. Additionally, women are screened at age 65 if they had a positive HPV test at age 60 (without referral).^1^ It is important to keep the age range of HPV screening limited to maintain the harms and benefits ratio of screening. For young women, harms could outweigh the benefits because of overdiagnosis (many HPV lesions at this age regress naturally) and because of the effect of vaccination (which leads to a decrease in benefits), while for older women, harms could outweigh the benefits when (pre)cancerous lesions are treated that would never have led to cancer death within the lifetime of the woman.^2^

However, the Dutch screening age range is narrower than that of most high‐income countries where screening often starts at age 25 or even earlier.^3^ Moreover, there are currently important developments for re‐evaluating if HPV screening should be done in a larger age range. For older women, life expectancy is increasing, and more life years will be saved per prevented cervical cancer death.^4^ For young women, there has been an increase in cervical cancer incidence observed over the past 10 years.^5^ Evidence shows that this effect does not only occur in the screening ages (i.e., >29 years), but also below age 30, and was already visible before the start of the hrHPV screening program in 2017. Also, an increase in both screen‐ and clinically detected cases (which is well documented in the Dutch pathology system) was reported, as well as an increase in all FIGO stages.^6^ Although the exact cause of the increase is still uncertain, this suggests that, instead of a change in the screening effect only, the increase may be explained by changes in the onset of disease (i.e., an increased risk of getting infected with HPV). Although this increase could be worrisome, it is important to consider whether this increase would outweigh the effects of vaccination in the coming years.

Therefore, the aim of this study is to use a microsimulation model to re‐evaluate potentially broader starting and ending ages of HPV screening in the Netherlands. The increase in cervical cancer incidence in women in younger birth cohorts will be incorporated into the model.

## 2 METHODS

For this analysis, we used a two‐step approach where we first calibrated a cohort effect into the MISCAN‐Cervix microsimulation model to incorporate the increase in cervical cancer incidence in young women.

### 2.1 MISCAN‐Cervix model

We used the microsimulation model MISCAN‐Cervix, described in Data S1A, to simulate a population of 100 million women born in 1962–2006 based on Dutch demographic, hysterectomy, and vaccination data.^7^, ^8^, ^9^, ^10^, ^11^ Women in the simulated population can acquire one or more high‐risk (hr) HPV infections during their life. The risk of acquiring an hrHPV infection is age‐ and type‐specific. An infection either clears or leads to the development of a pre‐invasive cervical lesion. These lesions can either regress or develop into invasive cervical cancer, classified in FIGO (International Federation of Gynecology and Obstetrics) stages 1A, 1B, 2, 3, and 4. Multiple infections can occur at the same time, which are independent of each other. Interventions such as hysterectomy, screening, and treatment can affect a woman's life history. Pre‐invasive stages (Cervical Intraepithelial Neoplasia [CIN] 1/2/3) and FIGO 1A cases can only be detected by screening, as these are assumed to be asymptomatic, whereas FIGO 1B or worse can also be clinically diagnosed. While pre‐invasive lesions can develop without an hrHPV infection (in which case they will always regress in our model), cervical cancer can only develop in the presence of an hrHPV infection. The HPV vaccination effect was calculated using the microsimulation model STDSIM.^12^ STDSIM is an individual‐based model that simulates a dynamic sexual network through which sexually transmitted infections, such as HPV, can be transmitted. With this model, the effect of vaccination on the prevalence of hrHPV can be simulated. The resulting effect is applied in MISCAN‐Cervix as a relative reduction in the hazard rate of HPV infections.

### 2.2 Calibration of cohort effects

Over the period 2014–2021 there has been an observed increase in cervical cancer incidence in the Netherlands.^5^ For the model to represent this increase properly, we recalibrated the model to include cohort effects using Dutch cancer incidence data from 2004 to 2021 by calendar year and age. The starting point for this calibration was the model described in Data S1A, which was not adjusted for this increase in incidence. Building on the parameter values described there, in the current calibration relative risks for disease onset were calibrated for four cohorts by using the genetic algorithm: 1978–1982, 1983–1988, and 1988–1992, 1993+ (see Data S1B for details on calibration settings).

### 2.3 Simulation cohorts and scenario's

We simulated four alternative screening strategies (starting in 2027, the start of the third hrHPV screening round in the Netherlands) where either the starting age or the ending age of screening was adjusted compared to the current Dutch screening program. For unvaccinated cohorts (1962–1992) we adjusted the ending age by adding a complete screening round at age 65 (strategy 1). We only consider a broader end age for unvaccinated cohorts since vaccinated cohorts are several decades away from reaching the age of 65, and in that time, the screening program will likely go through several changes, making the considerations for the ending age less relevant for these cohorts at this moment. Similarly, since no more women from fully unvaccinated cohorts will enter the screening program (Table S2 of Data S1C), we adjusted the starting age only for partly vaccinated cohorts (2002–2006) by adding a screening round at age 25 for either the full cohort (strategy 2), the vaccinated women in the cohort (strategy 2A) or the unvaccinated women in the cohort (strategy 2B).

### 2.4 Screening program

After a positive primary hrHPV‐test result, women are tested for genotypes HPV16/18/other high risk (OHR). Women with HPV16/18 and cytological abnormalities (atypical squamous cells of undetermined significance [ASC‐US] or higher) are referred to the gynecologist. Women with HPV‐OHR and high‐grade squamous intraepithelial lesion (HSIL) are also referred to the gynecologist. Other women with a positive hrHPV‐test result are invited for a repeat cytology test after 12 months. Attendance and adherence for the screening program are assumed to be 100%. In this way, we tailor the screening strategy to women who attend the screening program and we avoid unnecessary screening of these women. The assumptions for test characteristics, costs, and quality of life can be found in Table S3 of Data S1A and Table S1 of Data S1C.

### 2.5 Key outcomes

We analyzed harms and benefits of screening. For benefits of screening, we included cancers prevented, deaths prevented, life years gained (LYG) and quality‐adjusted life years (QALY) gained (compared to the current screening program). For harms of screening, we included the number of referrals, the number of unnecessary referrals, and the total costs of screening. We also included summary measures for efficiency and cost‐effectiveness. We looked at the efficiency of screening depicted by number needed to screen (NNS) to find one CIN2+ or CIN3+, number needed to refer (NNR) to find one CIN2+ or CIN3+, and positive predictive value (PPV) of the primary screening test for CIN2+ and CIN3+, and we looked at cost‐effectiveness as represented by the cost‐effectiveness ratio (CER) based on life years gained compared with the current screening program. We assumed a willingness‐to‐pay threshold of €50,000 per LYG. Outcomes are calculated from 2027 onwards. Costs and effects are discounted at a rate of 3%.

### 2.6 Sensitivity analysis

Since the future trajectory of the increase in cervical cancer incidence is uncertain, we performed a sensitivity analysis where we assume no increase in relative risk of disease onset by birth cohort. In other words, in this sensitivity analysis we assume that the steady cancer risk as observed in the past is a better representation of the future.

## 3 RESULTS

### 3.1 Calibration results

**FIGURE 1:**
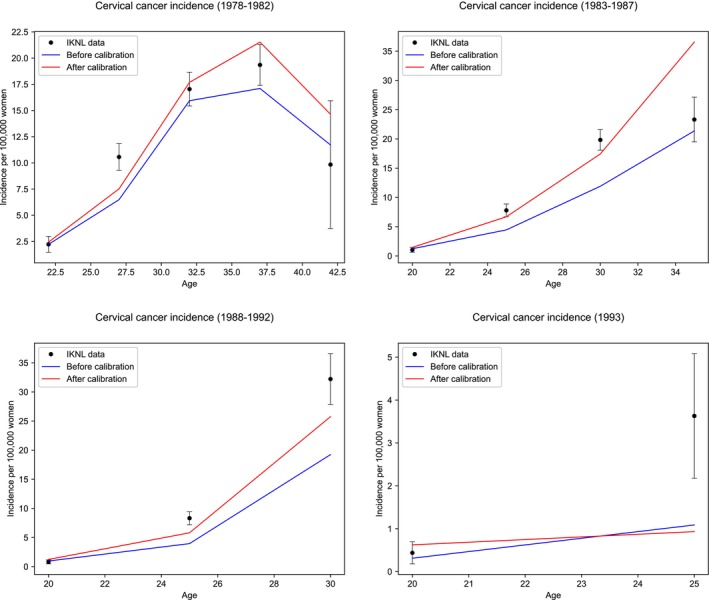
Cancer incidence as an average over 5 years for birth cohorts 1978–1982, 1983–1987, 1988–1992, and 1993+. The black data points are national cancer registry data with corresponding 95% confidence intervals. Blue and red lines are model outcomes before and after recalibration, respectively. CC, cervical cancer.

We found that the best fit for calibration was achieved with relative risks for onset of disease of 1.168 for birth cohort 1978–1982, 1.458 for 1983–1987, 1.373 for 1988–1992, and 1.371 for 1993 and later. The new calibration fit is presented in Figure 1.

### 3.2 Strategy comparison

#### 3.2.1 Base case

**FIGURE 2:**
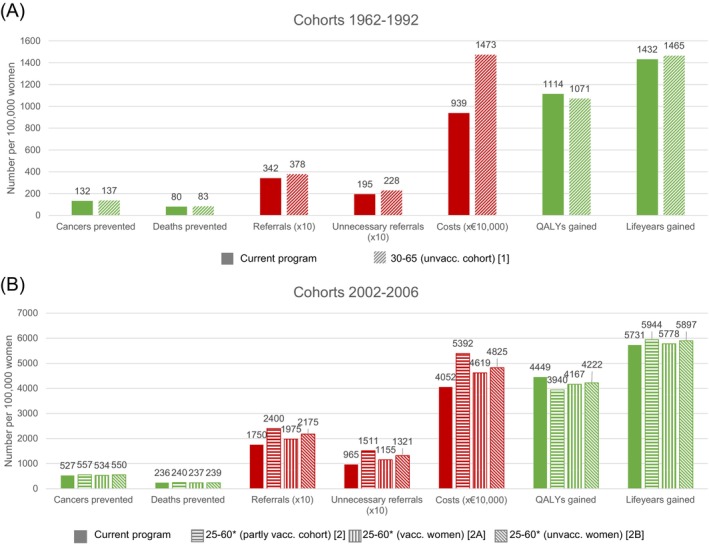
(Undiscounted) harms and benefits for unvaccinated cohorts (A) and partly vaccinated cohorts (B) compared to a situation without screening. *Women are additionally screened at age 65 if they tested HPV‐positive at age 60 and were not referred to a gynecologist.

For unvaccinated cohorts, most benefits increase slightly while harms increase substantially when screening at age 65 (Figure 2A). Deaths averted, cancers averted, and LYG increase by 4.0%, 3.5%, and 2.4%, respectively, whereas there is a decrease in QALYs gained of 3.8%. In contrast, the number of referrals, unnecessary referrals, and costs increase by 10.3%, 16.7%, and 57.0%, respectively.

For partly vaccinated cohorts, most benefits slightly increase by adding a screening at age 25 (deaths averted 2: +2.0%, 2A: +0.5%, 2B: +1.4%; cancers averted 2: +5.7%, 2A: +1.3%, 2B: +4.4% and LYG 2: +3.7%, 2A: +0.8%, 2B: +2.9%), while QALYs gained decrease compared to the current program (2: −11.4%, 2A: −6.3%, 2B: −5.1%) (Figure 2B). Furthermore, the harms increase substantially (referrals 2: +37.1%, 2A: +12.9%, 2B: +24.3%; unnecessary referrals 2: +56.6%, 2A: +19.7%, 2B: +36.9% and costs 2: +33.1%, 2A: +14.0%, 2B: +19.1%).

Efficiency decreases for all strategies when compared to the current program (Table 1). The PPVs of HPV+ decrease (CIN2+: 10.8% to 9.2%; CIN3+: 5.5% to 4.7%) when the ending age of screening is extended, and the NNS increases accordingly (CIN2+: 91.2 to 139.2; CIN3+: 177.9 to 271.4). Although the NNR also shows an increase, it is not as severe as the other efficiency measures (CIN2+: 2.3 to 2.5; CIN3+: 4.5 to 4.9). For screening at age 25, we also observe a decrease in the PPV of HPV+ (CIN2+: 13.6% to 10.6%–11.9%; CIN3+: 7.0% to 5.1%–6.0%) and an increase in NNS (CIN2+: 56.7 to 57.8–60.4; CIN3+: 110.7 to 118.8–126.8); moreover, the increase in NNR (CIN2+: 2.2 to 2.4–2.7; CIN3+: 4.3 to 4.8–5.7) is relatively large, especially when we decrease the starting age of screening for vaccinated women or for all women in vaccinated cohorts.

**TABLE 1:** Program efficiency and cost‐effectiveness comparison between strategies and the current program.

Nr.	Strategy	CIN2+ efficiency			CIN3+ efficiency			Cost‐effectiveness
		PPV HPV+^a^	NNS^b^	NNR^c^	PPV HPV+^a^	NNS^b^	NNR^c^	CER compared to current program
1962–1992	Current program	10.8%	91.2	2.3	5.5%	177.9	4.5	
1	30–65 (unvaccinated cohorts)	9.2%	139.2	2.5	4.7%	271.4	4.9	€275,096/LYG
2002–2006	Current program	13.6%	56.7	2.2	7.0%	110.7	4.3	
2	25–60 (mixed cohorts)	10.6%	60.4	2.7	5.1%	126.8	5.7	€163,362/LYG
2A	25–60 (vaccinated women)	11.9%	59.5	2.4	6.0%	118.8	4.8	€323,813/LYG
2B	25–60 (unvaccinated women)	11.8%	57.8	2.5	5.7%	119.1	5.2	€120,017/LYG

Abbreviations: CER, cost‐effectiveness ratio; HPV, human papillomavirus; NNR CIN2/3+, Number needed to refer to find one CIN2/3+; NNS CIN2/3+, Number needed to screen to find one CIN2/3+; PPV HPV, Positive predictive value of the primary HPV test.

^a^ Positive predictive value of the primary Human Papillomavirus (HPV) test. Calculated as the number of CIN2+/3+ lesions found after a positive HPV screening result divided by the total number of positive HPV tests.

^b^ Number needed to screen to find one CIN2+/3+ lesion. Calculated as the number of HPV screening tests performed divided by the number of CIN2+/3+ lesions found in the screening program.

^c^ Number needed to refer to find one CIN2+/3+ lesion. Calculated as the number of referrals to the gynecologist divided by the number of CIN2+/3+ lesions found in the screening program.

With a willingness‐to‐pay threshold of €50,000 per LYG, we find that screening at age 65 in unvaccinated cohorts is not cost‐effective (€275,096/LYG, Table 1). Screening at age 25 is not cost‐effective in vaccinated cohorts, including both vaccinated and unvaccinated women (2: €163,362/LYG, 2A: €323,813/LYG and 2B: €120,017/LYG).

### 3.3 Sensitivity analysis

The results of the sensitivity analysis without cohort effects for the relative risk of the onset of disease resemble the results of the base case, although the cost‐effectiveness ratio is higher in all cases. Screening unvaccinated cohorts at age 65 leads to a CER of €368,709/LYG, while screening partly vaccinated cohorts leads to a CER of €180,745/LYG (€144,680/LYG and €271,481/LYG, respectively, for unvaccinated women and vaccinated women). This means that none of the scenarios with broader age ranges are considered cost‐effective. The detailed results can be found in Data S1D.

## 4 DISCUSSION

For partly vaccinated cohorts, we found that starting HPV‐based screening earlier is not cost‐effective. On that account, we can conclude that, in general, the increase in cervical cancer incidence is nullified by the protecting effect of vaccination in these cohorts. Therefore, even though the increase in incidence is potentially worrisome in unvaccinated cohorts, HPV screening at a younger age in partly vaccinated cohorts likely leads to overscreening and should therefore be avoided. For unvaccinated cohorts, we also found that adding a screening for all women at age 65 would not be cost‐effective compared to the current program. This would lead to a small reduction in cervical cancer incidence and mortality but also decreases program efficiency. The number of referrals also increases, which leads to a relatively low increase in QALYs.

This study has a few limitations. Firstly, the cost‐effectiveness ratio has only been calculated for a very limited number of strategies. Increasing the number of strategies in the analysis might lead to different conclusions.^13^ Therefore, more research on the topic is necessary. Secondly, it should be taken into account that we recalibrated the increase in incidence using current cancer registry data, but it is uncertain how this trend will evolve over the coming years. Furthermore, the effect of Covid‐19 on the detection of cervical cancer in 2020–2021 could have played a role. To account for this source of uncertainty, we performed this analysis both with and without the cohort effects and came to a similar conclusion in both scenarios. Lastly, literature shows several factors that affect the effectiveness of screening. For example, there have been indications that latency of infections and differences in test characteristics by age play a role in the effectiveness of screening in older age groups.^14^ HPV latency could mean that the risk of progression is dependent on the duration of the infection, rather than on the age of the woman as assumed in our model. We did not take these factors into account, and as a result, the effect of screening in older age groups might have been somewhat over‐ or underestimated in this study.

As previously mentioned, the starting and ending ages of cervical cancer screening are important to optimize the screening program and are therefore widely studied. Previously, the optimal age range for screening in the Dutch setting has been studied by Van Rosmalen and colleagues.^15^ They investigated the cost‐effectiveness of several cytology‐based and HPV‐based screening programs and found that in unvaccinated cohorts, screening between the ages of 30 and 66 is considered a cost‐effective strategy at a cost‐effectiveness threshold of €50,000. The current study with updated data mostly supports this finding, although it does suggest that screening until the age of 65 for all women might not be cost‐effective compared to the current screening strategy. This difference can likely be explained by the comparatively longer screening intervals that were used in the study by Rosmalen and colleagues.

End age of hrHPV screening is starting to become an important topic of research. For the United States, there have been several studies to evaluate the end age for HPV screening.^16^, ^17^ Kim and colleagues used their simulation model to compare screening until age 65 (base case) with screening until age 70 or 75. It was concluded that screening efficiency is lower as the end age is increased, similar to what we have shown here. Landy and colleagues performed a study on empirical data and concluded that although more research is needed, exiting the screening program should be conditional on the test result from previous rounds. This is in line with the current screening guidelines in the Netherlands, which was the preferred strategy in the current study.

For the starting ages, many studies only consider cytology‐based screening under the age of 30.^18^, ^19^, ^20^ With this study, however, we aimed to investigate whether, with the current knowledge, extending HPV screening under the age of 30 is cost‐effective, and we found that it is most likely not cost‐effective in a partly vaccinated cohort. There are a few modelling studies that do consider the cost‐effectiveness of HPV screening under the age of 30, and they have similar findings. In the mathematical modelling study of Kim and colleagues, it was found that at a cost‐effectiveness threshold of $50,000 per LYG, HPV screening from age 25 is not considered cost‐effective.^21^ However, the study of Kim and colleagues only considered a fully vaccinated population. Naber and colleagues did consider partly vaccinated cohorts and investigated the effect of herd immunity on the cost‐effectiveness of screening while varying (among other things) the starting age.^22^ At none of the herd immunity levels was it cost‐effective to screen women at age 25.

In conclusion, screening women in unvaccinated cohorts at age 65 can lead to small health benefits, but does cause an increase in (unnecessary) referrals. It is therefore not a cost‐effective change to the screening program when using a willingness‐to‐pay threshold of €50,000 per LYG. Even after including an increased cohort effect for risk of disease onset, screening women in partly vaccinated cohorts at age 25 leads to little health benefits, while heavily increasing the number of (unnecessary) referrals. It is therefore not a cost‐effective change to the screening program.

## AUTHOR CONTRIBUTIONS

**Sylvia Kaljouw:** Conceptualization; formal analysis; investigation; methodology; visualization; writing – original draft; writing – review and editing. **Erik E. L. Jansen:** Conceptualization; methodology; supervision; visualization; writing – original draft; writing – review and editing. **Veerle J. C. Schevenhoven:** Formal analysis; investigation; software; validation; visualization; writing – review and editing. **Inge M. C. M. de Kok:** Conceptualization; funding acquisition; methodology; project administration; supervision; visualization; writing – original draft; writing – review and editing.

## FUNDING INFORMATION

This study was funded by the Dutch National Institute for Public Health and the Environment (Rijksinstituut voor Volksgezondheid en Milieu). The funding source (a government organisation) had no involvement in the study design, data collection, data analysis, interpretation of the data, writing of the report, or the decision to submit the paper for publication.

## CONFLICT OF INTEREST STATEMENT

All authors report receiving funding from the Dutch National Institute for Public Health and the Environment for the conduct of this study.

## Supporting information


**DATA S1.** Supporting Information.

## Data Availability

The data that support the findings of this study are available from the corresponding author upon reasonable request.
