# Supplementary material for: Should the age range of the Dutch hrHPV‐based cervical cancer screening program be broadened? A modelling study using cohort effects
Source: Int J Cancer. 2025 Apr 28;157(4):627–33. doi: 10.1002/ijc.35435 (PMC12178093; doi:10.1002/ijc.35435)
Supplement: Supplementary file 1 — DATA S1. Supporting Information. [file IJC-157-627-s001.pdf]

APPENDICES

Supplement to: S Kaljouw, EEJ Jansen, VJC Schevenhoven, IMCM de Kok Should the age range of the Dutch hrHPV-based cervical cancer screening program be broadened? A modelling study using cohort effects

Appendix A: Updated modelparameters…………………………………………………………..2

Appendix B: Calibration settings and results…………………………………………………….33

Appendix C: Assumptions for costs, QALYs lost and vaccination rate…………………………35

Appendix D: Results for sensitivity analysis without cohort effect……………………………..38

Appendix A: Model description of MISCAN-Cervix Netherlands (starting point for cohort effects calibration)

Much of this model description is the same as in the previous model description from Jansen, E. and colleagues.[1]

1. Model purpose

Cervical cancer screening can be used as a strategy to reduce both cervical cancer incidence and cervical cancer mortality.[2-4] Cervical cancer screening has been implemented in many different ways across Europe. To assess the costs and effects of such screening programmes one could monitor the existing programmes or set up trials to evaluate different screening strategies. However, setting up large trials is expensive, needs a long follow-up time and might have ethical concerns. Also, the outcomes of such trials will be dependent on several factors, which might be different across countries, so the results might not be applicable to another country.

MISCAN-Cervix is a microsimulation model that is able to simulate a hypothetical population, including the development of cervical cancer. In these simulations, different screening strategies can be applied to quantify the effects of screening in this population.[5] Because the population characteristics in the model and their background risk for cervical cancer can be tailored to those of a specific country, it is possible to make country-specific estimations of costs and effects for different screening strategies. In this study, the model was set to represent the Dutch situation.

The three main aims of MISCAN-Cervix are:

1. To quantify the long-term harms, benefits, costs and cost-effectiveness of primary prevention and cervical cancer screening strategies.
2. To compare screening strategies, allowing the user to improve existing screening programmes as well as advising countries on the effects of implementing a cervical cancer screening programme.
3. To quantify the effects of removing barriers to screening, which can be used to prioritize which barriers should be removed first.

2. Model overview

The Microsimulation Screening Analysis (MISCAN) program was first developed in 1985 by the Department of Public Health of Erasmus MC University Medical Center in The Netherlands to evaluate

the effects of screening on disease.[5] Since then, the MISCAN program has been used to quantify the effects of screening for cancers of the breast, colon, cervix, prostate, and lung.[6-10]

MISCAN-Cervix, coded in Python (previously in Borland Delphi 7), is a stochastic, semi-Markov microsimulation model. In a microsimulation model, individuals are simulated one at a time instead of as proportions of a cohort. The advantage of this is that new events can be dependent on past events of that individual. The model is stochastic, which means that sequences of events are simulated by drawing from distributions of probabilities and durations instead of using fixed values. Therefore, the outcomes of the model are subject to random variation.

2.1 Model description

Figure 1 shows the basic structure of MISCAN-Cervix. The program consists of three main parts:

1. Demography part
2. Natural history part
3. Screening part

Input data for these parts is processed by the MISCAN program to generate individual life histories of women in a population. The program can both simulate a situation without screening and a situation with the selected screening programme. The difference between the outcomes of those two scenarios is considered as the effect of screening. All three parts are described further in the upcoming sections.

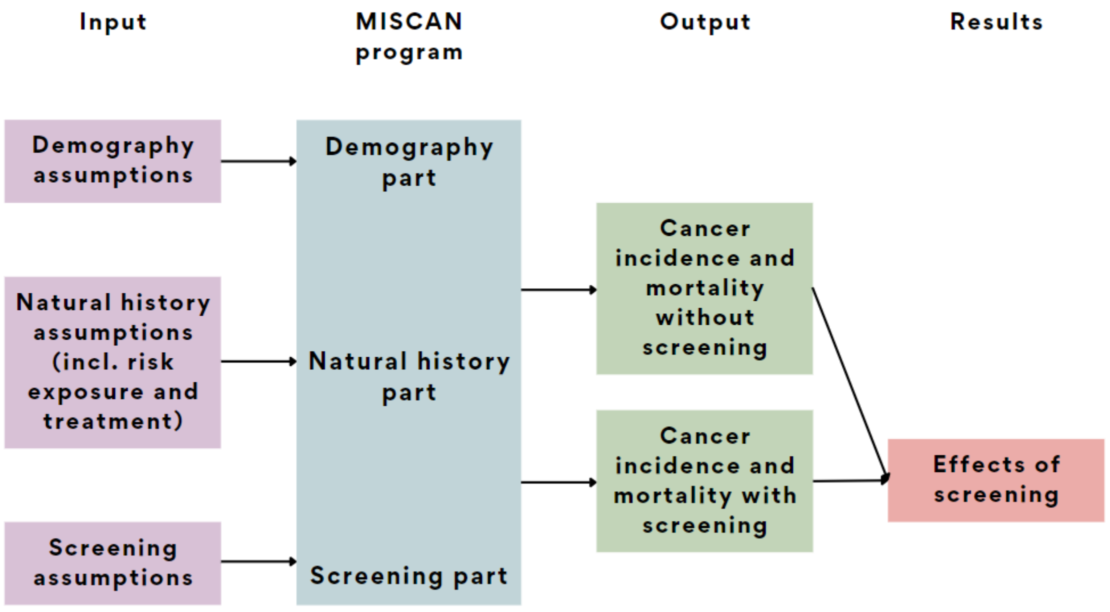

Figure 1 Basic structure of MISCAN-Cervix

2.2 Demography part

MISCAN-Cervix simulates a female population. A specific life history is generated for each woman, including a date of birth and a date of death from other causes than cervical cancer. Simulated women also have a probability to have a hysterectomy, for reasons other than cervical cancer, depending on the age and the birth year of the woman. In the model, women cannot become older than 100 years of age.

2.3 Natural history part

Figure 2 shows the natural history structure of MISCAN-Cervix. In the static MISCAN model, several health states with corresponding durations and transition probabilities are defined.

Each woman starts at the 'Normal' state and has an age-specific risk of acquiring one or multiple infections with a high-risk type of the human papillomanvirus (hrHPV). These hrHPV types can cause cancer and are detectable by the hrHPV-test. The model distinguishes between four categories of hrHPV types, based on their likeliness to cause cancer and efficacy of different vaccines against these types:

1. HPV-16
2. HPV-18
3. High risk types that are covered in the nonavalent vaccine (HPV-31/33/45/52/58)
4. Other high risk HPV types (HPV-35/39/51/56/59/66/68)

An acquired hrHPV infection will most likely regress, but may progress to a pre-invasive cervical intraepithelial neoplasia (CIN) grade 1, which may in turn progress sequentially to CIN grade 2 and 3. Regression probabilities of pre-invasive lesions are dependent on age and lesion grade. A woman might also develop CIN lesions without the presence of hrHPV, although hrHPV-negative lesions will never progress to cancer.[11] A woman can have multiple lesions and hrHPV infections simultaneously (max 200 in the model), which may or may not progress independent of each other.[12]

A CIN3 lesion may progress to cervical cancer, which is modeled in five different stages according to the Fédération Internationale de Gynécologie et d'Obstétrique (FIGO) classification: FIGO stages 1A, 1B, 2, 3 and 4. Cancers classified as FIGO stage 1B and higher may be detected clinically (i.e. because of symptoms) before progressing to a higher stage. Screening may detect all pre-invasive and invasive lesions. Survival probabilities and durations until cervical cancer death depend on the stage and on the age at which the cancer is detected. A death is only counted as a cervical cancer death if the woman dies from cervical cancer before dying from other causes.

During the development of disease, a woman might die of other causes than cervical cancer at any moment. A woman might also have a hysterectomy for other reasons than cervical cancer at any

moment, removing all prevalent hrHPV infections and CIN lesions. Women who have had a hysterectomy will no longer be at risk for acquiring an hrHPV infection or CIN lesion and will not be invited for screening any more.

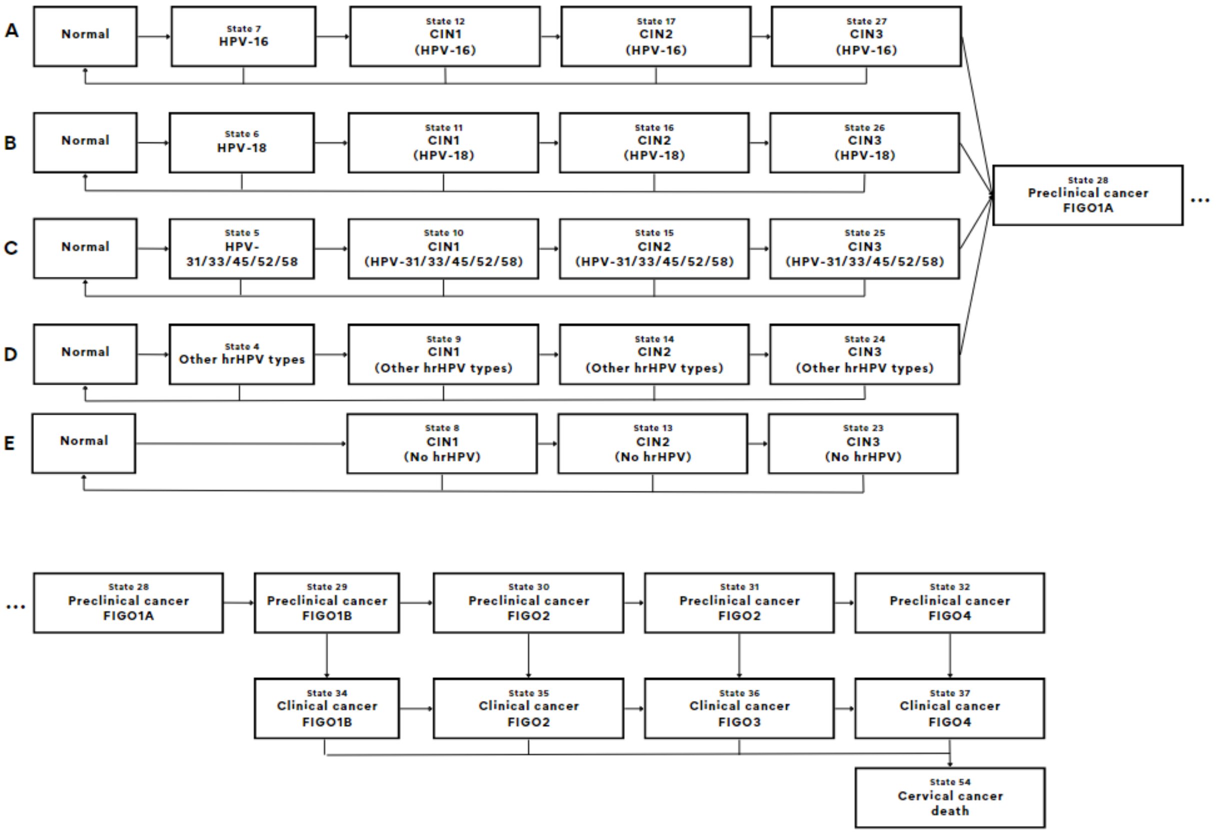

Figure 2 The natural history structure of MISCAN-Cervix.

(hr)HPV = (high-risk) human papillomavirus; CIN = cervical intraepithelial neoplasia; FIGO = International Federation of Gynaecology and Obstetrics

If a woman acquires an hrHPV infection, the prevalence of disease is added to the original life history of this woman generated in the demography part, resulting in a life history with disease, as shown in Figure 3. In this case, the woman acquires two hrHPV infections. The first infection will progress to a CIN2 before the infection clears and the lesion regresses, while the second infection will progress to cervical cancer and cause a cervical cancer death. In the bottom line the original life history is combined with the natural history of cervical cancer. Here we see that the moment of death from cervical cancer occurs before the moment of death from other causes, altering the original moment of death. The difference between the original moment of death from other causes and the moment of death from cervical cancer is the number of life years lost due to cervical cancer.

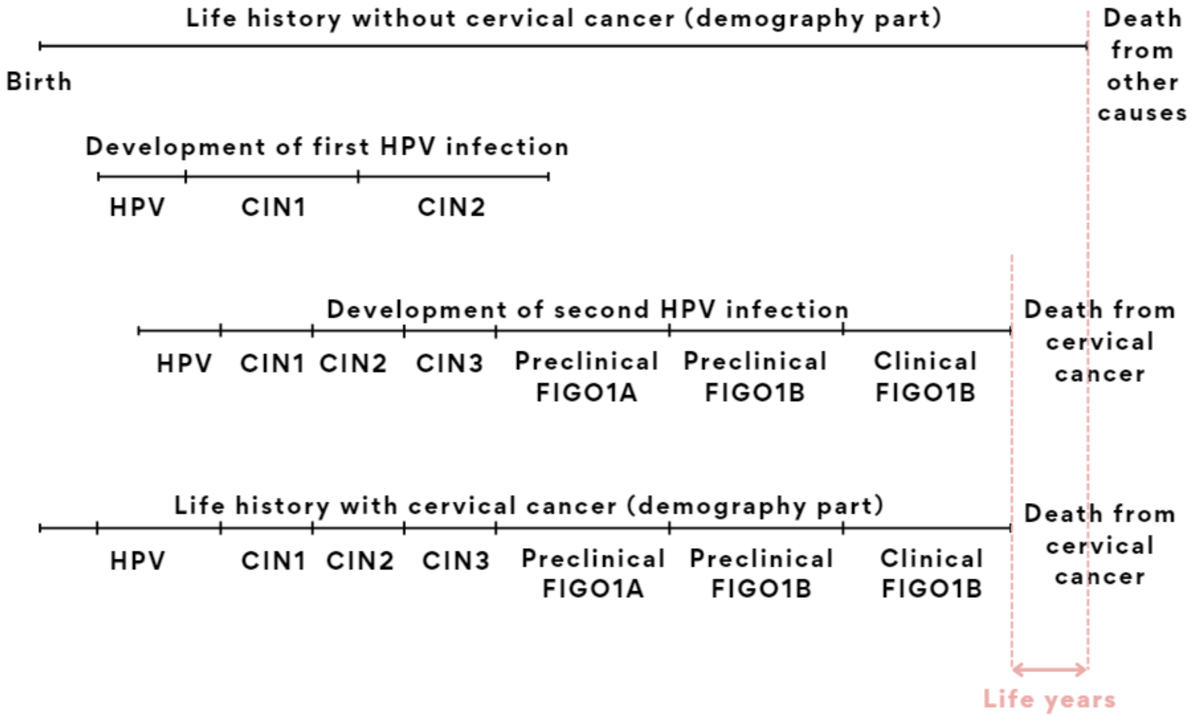

Figure 3 Life histories with and without disease.

HPV = human papillomavirus; CIN = cervical intraepithelial neoplasia; FIGO = International Federation of Gynaecology and Obstetrics.

2.4 Screening part

In the screening part of MISCAN-Cervix, screening strategies and screening behavior are simulated. Women can be invited to participate in screening at specified ages. Depending on the test used and the highest prevalent lesion of a woman at the moment of the screening test, there is a probability of a positive test result. If the screening test is positive, a woman will be referred for either a triage test or a referral to colposcopy, depending on the applied strategy. When a woman is referred to colposcopy, all prevalent CIN lesions will be diagnosed and successfully removed/treated. In practice, low grade pre-invasive lesions (e.g. CIN1) might not be treated directly, but as these women will be monitored regularly and treated if the lesion progresses, these low grade lesions are considered as removed in the model.

As screen-detected cancers tend to have a better stage-specific survival than clinically detected cancers, detection of cervical cancer by screening in the model may prevent death from cervical cancer. However, if the death from cervical cancer is not prevented, the duration from the moment of detection until the moment of death from cervical cancer will not be different from clinically detected cancers.

The effect of screening on the life history of a woman is shown in Figure 4. In this case, the woman attends a screening intervention, removing the prevalent CIN lesions. The second hrHPV infection will not lead to cancer anymore and therefore, the woman will die at the original moment of death from

other causes. The difference between the moment she would have died from cervical cancer and the moment she will now die of other causes is the screen effect and can be quantified as the number of life years gained.

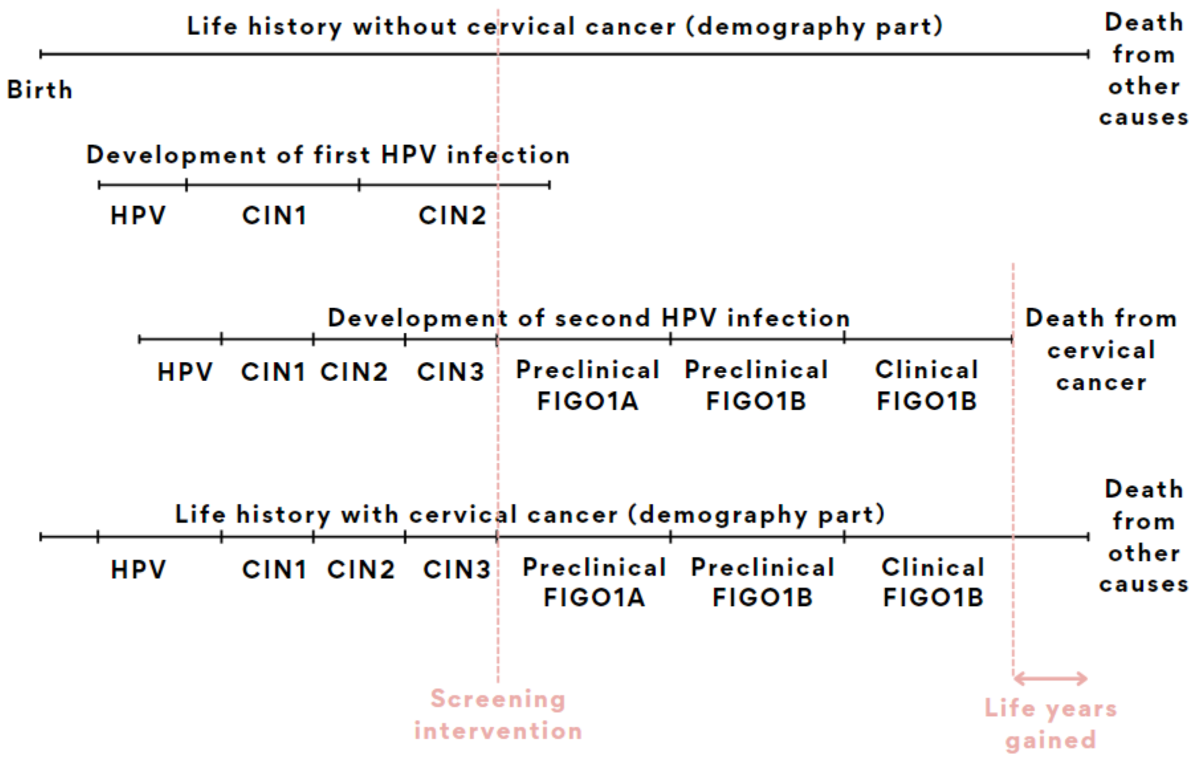

Figure 4 Life history with screening.

HPV = human papillomavirus; CIN = cervical intraepithelial neoplasia; FIGO = International Federation of Gynaecology and Obstetrics.

3. Model output

After the simulation is complete, several types of outcomes can be reported by the program, such as:

3.1 Demographic or epidemiological outputs

1. Total life-years lived
2. Cervical cancer incidence rates by age group
3. Cervical cancer mortality rates by age group
4. Cervical cancer incidence counts by age group
5. Cervical cancer mortality counts by age group
6. Number of cervical cancer deaths per 100 000 women simulated lifelong

3.2 Screening outputs

1. Number of screen tests and triage tests by age group
2. Number of false positive referrals to colposcopy
3. Number of referrals to colposcopy by lesion grade and by age

4. Cervical cancer mortality reduction due to screening
5. Number of tests needed to prevent 1 cervical cancer incidence or death
6. Number of colposcopies needed to prevent 1 cervical cancer incidence or death
7. Life-years gained by screening

3.3 Undiscounted and discounted costs and QALY's

1. Costs of screening
2. Costs of diagnosis and treatment of disease
3. Costs of palliative care
4. Life-years gained compared to no screening scenario
5. QALY's gained compared to a no screening scenario
6. Costs/life-year gained
7. Costs/QALY gained

4. Structural model assumptions

Several assumptions have to be made for each part of the model, either because data needed to directly calculate inputs are lacking or because complex processes needed to be simplified in order to be able to model them.

Making assumptions introduces uncertainty about the model outcomes. Therefore, model validation is used to test the model assumptions. During model validation, the model is set to simulate a 'real-life' situation, for example, a cohort study. The model outputs can then be compared with the outcomes of the real-world study that the model was trying to simulate. If differences in results cannot be explained by external reasons, such as underreporting of cause-specific deaths, the model parameter values are re-estimated. If this re-estimation of model parameters does not lead to a satisfying result, the model assumptions are reconsidered. Also, sensitivity analyses are usually performed in which assumptions are varied which might be crucial for the conclusion of an analysis, to see if the conclusion still holds with these alternative assumptions.

4.1 Demography assumptions

The following demography assumptions were made, which affect the characteristics of the population without the presence of cervical cancer.

- One cohort is simulated with one life table. All women are born at the same time, but will die from cervical cancer or from other causes at different moments in time.
- In the model it is assumed that death from cervical cancer is independent from death from other causes. Whichever comes first determines the actual moment of death.

4.2 Natural history assumptions

Many characteristics of the natural history of cervical cancer cannot be observed because the disease starts to develop unnoticed. Once a diagnosis is made, it is in most cases unethical not to intervene. Therefore, assumptions have to be made about the natural history of cervical cancer. These assumptions are based on expert opinion or derived from observed data such as detection rates. For an overview of the natural history part of the model, please see the natural history section of the model overview.

Human Papillomavirus

- Each woman has an age-specific risk of acquiring hrHPV infections.
- A woman can acquire multiple hrHPV infections during her lifetime, and these hrHPV infections may be present at the same time. The progression of these lesions are modelled independently, there is no interaction.
- If vaccination is introduced, there will be an age-specific relative reduction of the age-specific risk of acquiring hrHPV infections, depending on the vaccination type and vaccination coverage.
- Most hrHPV infections will clear naturally before progressing to cervical intraepithelial neoplasia (CIN).
- As described in the natural history section of the model overview, the model distinguishes four categories of hrHPV genotypes. The duration of hrHPV-infections and subsequent CIN lesions are assumed equal for all genotypes, but the duration of an hrHPV-infection increases with age. However, the progression probabilities from all pre-invasive health states are different between all genotypes and are dependent on age as well.
- If a woman has a hysterectomy because of cervical cancer or for other reasons than cervical cancer, all hrHPV infections are considered removed as well. No new hrHPV infections can be acquired.

Cervical Intraepithelial Neoplasia (CIN)

- Most CIN1 lesions will develop from an hrHPV infection.
- Each woman has an age-specific risk of developing a CIN1 lesion in the absence of hrHPV.
- Progression probabilities for CIN lesions depend on lesion grade, age and hrHPV genotype. Most CIN1 lesions will clear before progression to CIN2. Those that progress to CIN2 will mostly clear before progression to CIN3. hrHPV-negative CIN3 will never progress to cancer.[11]
- If a woman has a hysterectomy because of cervical cancer or for other reasons than cervical cancer, all CIN lesions are considered removed as well. No new CIN lesions can be developed.

Cervical cancer

- Cervical cancer always develops following a hrHPV-positive CIN3 lesion.
- After the detection of cervical cancer, the woman has a hysterectomy. Therefore, we do not assume any possibility of having a recurrent cervical cancer.

- Preclinical FIGO1A does not cause symptoms yet and will therefore never be clinically detected. Preclinical FIGO1B or higher stages can be detected clinically in the absence of screening or can progress to a higher cancer stage (Figure 2).
- Durations of the different cancer stages do not depend on age or genotype.
- Once a lesion has become cancer, progression probabilities to higher cancer stages depend on age, but are equal across genotypes.
- Clinically detected cervical cancer can either be cured or cause cervical cancer death. The probability to die from cervical cancer is dependent on the cancer stage and the age of the woman.
- If the woman is cured she will stay in the cancer state until death of other causes. If the woman is not cured, she will die of cervical cancer within a maximum of 10 years after diagnosis.

Hysterectomy

- Women who do not have cervical cancer have an age-specific probability of getting a hysterectomy for reasons other than cervical cancer.
- A hysterectomy is assumed to remove all prevalent hrHPV infections and CIN lesions.
- Women who have had a hysterectomy will no longer acquire new hrHPV infections or develop new CIN lesions and will no longer be invited for screening tests.

4.3 Screening assumptions

Several assumptions have to be made regarding the performance of the screening tests, the consequences of colposcopy and the screening behavior of the women. For an overview of the screening part of the model, please see the screening part section of the model overview.

Terminology

- The screening strategy of an organised screening programme determines between which ages women are invited for screening, with which interval they are invited, which primary test is performed and which triage tests are performed after a positive primary test.
- A primary test is the initial screening test a woman is invited to. Based on the result of this test, the woman will be referred to colposcopy or triage testing.
- The primary test can either be cytology (checking for abnormal cells), hrHPV-test (checking for the presence of hrHPV) or a co-test, which is a combination of both.
- A triage test is a screening test that is performed after a woman has had a positive primary screening test, but before the decision is made whether or not to refer her for a colposcopy (e.g. a cytology test after a positive primary hrHPV-test). The triage test can be performed either directly after the primary test, or after a waiting period of several months or years, depending on the screening strategy.
- A colposcopy is a diagnostic exam by a gynaecologist to determine the presence of disease. This might include taking a biopsy.

Performance of the screening tests

- The probability of having a positive test result depends on the lesion grade and the hrHPV status of the woman for both cytology and the hrHPV-test.
- No differences in test characteristics are assumed for different hrHPV genotypes, both for cytology and the hrHPV-test.
- Systematic positive and systematic negative test results over time are possible for cytology for certain women, infections or lesions.

Screening behaviour

- Women invited to screening can either attend or not attend the primary screening test. The probability to attend is dependent on age. If a woman attends, she will do so exactly at the invited age.
- If a woman attends the primary test and is referred to triage testing or colposcopy, she might not adhere to this referral.
- If a scenario is simulated where the age ranges of the screening programme are extended compared to the current target ages, women in those newly targeted age groups will attend with the same probability as the closest age group that is invited in the current screening programme.

Colposcopy

- When a woman is referred to colposcopy, all prevalent CIN lesions will be diagnosed and removed/treated.
- Colposcopy is 100% accurate and will show the highest prevalent lesion.
- Women with a prevalent hrHPV infection but without a prevalent CIN will not be treated. The hrHPV infection may still progress to CIN after the colposcopy.
- As screen-detected cancers tend to have a better stage-specific survival than clinically detected cancers, detection of cervical cancer by screening in the model may prevent death from cervical cancer. However, if the death from cervical cancer is not prevented, the duration until death from cervical cancer will not be different from clinically detected cancers.

5. Model parameters

Next to the assumptions on the structure of the model, calculations or assumptions have to be made to determine the exact values of certain model parameters. In this section, we will describe the type of parameters that serve as inputs for the model and how the values of these parameters are determined. The parameters are categorized by the part of the model they belong to. Please see the model overview section above for more information about the model parts. Some parameters were calibrated. Please see the calibration section for more information about the calibration process.

5.1 Demographic part

The model simulates a cohort of women born in the years 1962-1992 and a cohort of women born in the years 2002-2006. The first cohort of women did not qualify for the hrHPV vaccination and the second

cohort did. Each woman has a probability to die at each age as reported by Statistics Netherlands in The Netherlands for Dutch women in 2021.[13] Dutch age-specific hysterectomy probabilities by age were also obtained from the CBS for the most recent year available (2010).[14]

5.2 Natural history part

Background risk

The age-specific background risk for acquiring a hrHPV infection is calibrated to the hrHPV-prevalence as observed in the Dutch screening programme (see calibration section). The age-specific distribution of hrHPV infections over the four categories of genotypes is based on Coupe et al. 2008.[15] The background risk for acquiring a hrHPV infection for the 10% of the population that never attends screening ('never attenders') was previously found to be higher than that risk for the remaining 90% of the population that attends screening. In a previous calibration, the relative background risk of never attenders was estimated to be 2.6 compared to the rest of the population. In this calibration the background risk is drawn from an Exponential distribution with the previously calibrated means and a newly calibrated variance of 2.0.

Progression probabilities of pre-invasive lesions

The age-specific probabilities that an hrHPV infection will progress to CIN1, CIN2, CIN3 or cancer are estimated during the calibration process using, among other sources, detection rates of the Dutch screening programme as a calibration target (see calibration section).

Table 1: Transition probabilities (regression and progression) per HPV type, age group and current state, as defined in MISCAN-Cervix.

HPV type	Age*	Regression From	To	Probability	Progression From	To	Probability
HPV 16	15	HPV 16	No HPV	0.853	HPV 16	CIN 1	0.147
HPV 16	25	HPV 16	No HPV	0.925	HPV 16	CIN 1	0.075
HPV 16	35	HPV 16	No HPV	0.336	HPV 16	CIN 1	0.664
HPV 16	50	HPV 16	No HPV	0.153	HPV 16	CIN 1	0.847
HPV 16	75	HPV 16	No HPV	0.120	HPV 16	CIN 1	0.880
HPV 18	15	HPV 18	No HPV	0.831	HPV 18	CIN 1	0.169
HPV 18	25	HPV 18	No HPV	0.909	HPV 18	CIN 1	0.091
HPV 18	35	HPV 18	No HPV	0.304	HPV 18	CIN 1	0.696
HPV 18	50	HPV 18	No HPV	0.117	HPV 18	CIN 1	0.883
HPV 18	75	HPV 18	No HPV	0.120	HPV 18	CIN 1	0.880
HPV 9V	15	HPV 9V	No HPV	0.860	HPV 9V	CIN 1	0.140
HPV 9V	25	HPV 9V	No HPV	0.929	HPV 9V	CIN 1	0.071
HPV 9V	35	HPV 9V	No HPV	0.346	HPV 9V	CIN 1	0.654
HPV 9V	50	HPV 9V	No HPV	0.164	HPV 9V	CIN 1	0.836

HPV 9V	75	HPV 9V	No HPV	0.120	HPV 9V	CIN 1	0.880
HPVOHR	15	HPVOHR	No HPV	0.860	HPVOHR	CIN 1	0.140
HPVOHR	25	HPVOHR	No HPV	0.929	HPVOHR	CIN 1	0.071
HPVOHR	35	HPVOHR	No HPV	0.347	HPVOHR	CIN 1	0.653
HPVOHR	50	HPVOHR	No HPV	0.164	HPVOHR	CIN 1	0.836
HPVOHR	75	HPVOHR	No HPV	0.120	HPVOHR	CIN 1	0.880
HPV 16	20	CIN 1	HPV 16	0.556	CIN 1	CIN 2	0.444
HPV 16	35	CIN 1	HPV 16	0.038	CIN 1	CIN 2	0.962
HPV 16	50	CIN 1	HPV 16	0.519	CIN 1	CIN 2	0.481
HPV 16	65	CIN 1	HPV 16	0.869	CIN 1	CIN 2	0.131
HPV 18	20	CIN 1	HPV 18	0.880	CIN 1	CIN 2	0.120
HPV 18	35	CIN 1	HPV 18	0.741	CIN 1	CIN 2	0.259
HPV 18	50	CIN 1	HPV 18	0.870	CIN 1	CIN 2	0.130
HPV 18	65	CIN 1	HPV 18	0.965	CIN 1	CIN 2	0.035
HPV 9V	20	CIN 1	HPV 9V	0.736	CIN 1	CIN 2	0.264
HPV 9V	35	CIN 1	HPV 9V	0.427	CIN 1	CIN 2	0.573
HPV 9V	50	CIN 1	HPV 9V	0.714	CIN 1	CIN 2	0.286
HPV 9V	65	CIN 1	HPV 9V	0.922	CIN 1	CIN 2	0.078
HPVOHR	20	CIN 1	HPVOHR	0.877	CIN 1	CIN 2	0.123
HPVOHR	35	CIN 1	HPVOHR	0.732	CIN 1	CIN 2	0.268
HPVOHR	50	CIN 1	HPVOHR	0.866	CIN 1	CIN 2	0.134
HPVOHR	65	CIN 1	HPVOHR	0.964	CIN 1	CIN 2	0.036
NoHPV	20	CIN 1	No HPV	0.762	CIN 1	CIN 2	0.238
NoHPV	35	CIN 1	No HPV	0.485	CIN 1	CIN 2	0.515
NoHPV	50	CIN 1	No HPV	0.743	CIN 1	CIN 2	0.257
NoHPV	65	CIN 1	No HPV	0.930	CIN 1	CIN 2	0.070
HPV 16	20	CIN 2	CIN 1	0.518	CIN 2	CIN 3	0.482
HPV 16	35	CIN 2	CIN 1	0.459	CIN 2	CIN 3	0.541
HPV 16	50	CIN 2	CIN 1	0.766	CIN 2	CIN 3	0.234
HPV 16	65	CIN 2	CIN 1	0.704	CIN 2	CIN 3	0.296
HPV 18	20	CIN 2	CIN 1	0.815	CIN 2	CIN 3	0.185
HPV 18	35	CIN 2	CIN 1	0.792	CIN 2	CIN 3	0.208
HPV 18	50	CIN 2	CIN 1	0.910	CIN 2	CIN 3	0.090
HPV 18	65	CIN 2	CIN 1	0.886	CIN 2	CIN 3	0.114
HPV 9V	20	CIN 2	CIN 1	0.657	CIN 2	CIN 3	0.343
HPV 9V	35	CIN 2	CIN 1	0.615	CIN 2	CIN 3	0.385
HPV 9V	50	CIN 2	CIN 1	0.833	CIN 2	CIN 3	0.167
HPV 9V	65	CIN 2	CIN 1	0.789	CIN 2	CIN 3	0.211
HPVOHR	20	CIN 2	CIN 1	0.729	CIN 2	CIN 3	0.271
HPVOHR	35	CIN 2	CIN 1	0.696	CIN 2	CIN 3	0.304
HPVOHR	50	CIN 2	CIN 1	0.868	CIN 2	CIN 3	0.132
HPVOHR	65	CIN 2	CIN 1	0.833	CIN 2	CIN 3	0.167
NoHPV	20	CIN 2	CIN 1	0.609	CIN 2	CIN 3	0.391

NoHPV	35	CIN 2	CIN 1	0.561	CIN 2	CIN 3	0.439
NoHPV	50	CIN 2	CIN 1	0.810	CIN 2	CIN 3	0.190
NoHPV	65	CIN 2	CIN 1	0.760	CIN 2	CIN 3	0.240
HPV 16	20	CIN 3	CIN 2	0.930	CIN 3	CC	0.070
HPV 16	35	CIN 3	CIN 2	0.882	CIN 3	CC	0.118
HPV 16	50	CIN 3	CIN 2	0.865	CIN 3	CC	0.135
HPV 16	65	CIN 3	CIN 2	0.090	CIN 3	CC	0.910
HPV 18	20	CIN 3	CIN 2	0.561	CIN 3	CC	0.439
HPV 18	35	CIN 3	CIN 2	0.254	CIN 3	CC	0.746
HPV 18	50	CIN 3	CIN 2	0.147	CIN 3	CC	0.853
HPV 18	65	CIN 3	CIN 2	0.090	CIN 3	CC	0.910
HPV 9V	20	CIN 3	CIN 2	0.970	CIN 3	CC	0.030
HPV 9V	35	CIN 3	CIN 2	0.949	CIN 3	CC	0.051
HPV 9V	50	CIN 3	CIN 2	0.942	CIN 3	CC	0.058
HPV 9V	65	CIN 3	CIN 2	0.090	CIN 3	CC	0.910
HPVOHR	20	CIN 3	CIN 2	0.981	CIN 3	CC	0.019
HPVOHR	35	CIN 3	CIN 2	0.968	CIN 3	CC	0.032
HPVOHR	50	CIN 3	CIN 2	0.963	CIN 3	CC	0.037
HPVOHR	65	CIN 3	CIN 2	0.090	CIN 3	CC	0.910
No HPV	20	CIN 3	CIN 2	1.000	CIN 3	CC	0.000**
No HPV	35	CIN 3	CIN 2	1.000	CIN 3	CC	0.000**
No HPV	50	CIN 3	CIN 2	1.000	CIN 3	CC	0.000**
No HPV	65	CIN 3	CIN 2	1.000	CIN 3	CC	0.000**

hrHPV = high-risk human papillomavirus; CIN = cervical intraepithelial neoplasia; CC = cervical cancer; HPV 9V = HPV-31/33/45/52/58; HPVOHR = HPV-35/39/51/56/59/66/68

* Remaining ages are linearly interpolated based on the values in the table.
** CIN 3 lesions can never transition to cervical cancer without an HPV infection

Probabilities of clinical detection of cancer

The age-specific probability that a FIGO1B, FIGO2 or FIGO3 cancer will be clinically detected before it progresses to higher cancer stage is based on the calibration process during which the cancer stage distribution was the main calibration target. FIGO1A is assumed not to give symptoms, so it will not be clinically detected, whilst FIGO4 cannot progress to a higher cancer stage, so will always be clinically detected at that stage.

Duration of health states

Most health states in the model have a duration, before transition to a next health state, that is drawn from a Weibull distribution. The duration is drawn from the distribution once the progression probabilities have determined whether the lesion progresses or regresses from the current state. Most

of these distributions have a Weibull shape parameter of 1, making them an exponential distribution. The duration of an hrHPV infection is the same for all four genotypes, but increases with age. The mean and shape of the Weibull distribution from which these durations are drawn are calibrated. The duration of clinical cervical cancer (states 34-37) and death from cervical cancer, if a woman is not cured, is assumed to be less than ten years and based on stage-specific survival data from the Dutch cancer registry (NKR).

Table 2 Durations of health states

Transition number*	From state*	To state*	Ages	Mean duration (years)	Weibull shape	Source
1	4 HPV-OHR	1 Normal	0-29	1.86	0.78	Calibration
			30-39	2.36	1.49	
			40-49	4.91	1.61	
			50-100	12.11	150.28	
2	4 HPV-OHR	9 HPV-OHR CIN1	0-29	1.86	0.78	Calibration
			30-39	2.36	1.49	
			40-49	4.91	1.61	
			50-100	12.11	150.28	
3	5 HPV-9V	1 Normal	0-29	1.86	0.78	Calibration
			30-39	2.36	1.49	
			40-49	4.91	1.61	
			50-100	12.11	150.28	
4	5 HPV-9V	10 HPV-9V CIN1	0-29	1.86	0.78	Calibration
			30-39	2.36	1.49	
			40-49	4.91	1.61	
			50-100	12.11	150.28	
5	6 HPV-18	1 Normal	0-29	1.86	0.78	Calibration
			30-39	2.36	1.49	
			40-49	4.91	1.61	
			50-100	12.11	150.28	
6	6 HPV-18	11 HPV-18 CIN1	0-29	1.86	0.78	Calibration
			30-39	2.36	1.49	
			40-49	4.91	1.61	
			50-100	12.11	150.28	
7	7 HPV-16	1 Normal	0-29	1.86	0.78	Calibration
			30-39	2.36	1.49	
			40-49	4.91	1.61	
			50-100	12.11	150.28	
8	7 HPV-16	12 HPV-16 CIN1	0-29	1.86	0.78	Calibration
			30-39	2.36	1.49	
			40-49	4.91	1.61	
			50-100	12.11	150.28	
9	8 NoHPV CIN1	1 Normal	0-100	1.5	1	[16]
10	8 NoHPV CIN1	13 NoHPV CIN2	0-100	1.5	1	[16]
11	9 HPV-OHR CIN1	1 Normal	0-100	1.5	1	[16]
12	9 HPV-OHR CIN1	14 HPV-OHR CIN2	0-100	1.5	1	[16]
13	10 HPV-9V CIN1	1 Normal	0-100	1.5	1	[16]
14	10 HPV-9V CIN1	15 HPV-9V CIN2	0-100	1.5	1	[16]
15	11 HPV-18 CIN1	1 Normal	0-100	1.5	1	[16]
16	11 HPV-18 CIN1	16 HPV-18 CIN2	0-100	1.5	1	[16]
17	12 HPV-16 CIN1	1 Normal	0-100	1.5	1	[16]
18	12 HPV-16 CIN1	17 HPV-16 CIN2	0-100	1.5	1	[16]
19	13 NoHPV CIN2	1 Normal	0-100	2	1	[16, 17]
20	13 NoHPV CIN2	23 NoHPV CIN3	0-100	2	1	[16, 17]
21	14 HPV-OHR CIN2	1 Normal	0-100	2	1	[16, 17]
22	14 HPV-OHR CIN2	24 HPV-OHR CIN3	0-100	2	1	[16, 17]
23	15 HPV-9V CIN2	1 Normal	0-100	2	1	[16, 17]

24	15 HPV-9V CIN2	25 HPV-9V CIN3	0-100	2	1	16, 17
25	16 HPV-18 CIN2	1 Normal	0-100	2	1	16, 17
26	16 HPV-18 CIN2	26 HPV-18 CIN3	0-100	2	1	16, 17
27	17 HPV-16 CIN2	1 Normal	0-100	2	1	16, 17
28	17 HPV-16 CIN2	27 HPV-16 CIN3	0-100	2	1	16, 17
29	23 NoHPV CIN3	1 Normal	0-100	5.7	0.84	Calibration
30	24 HPV-OHR CIN3	1 Normal	0-100	5.7	0.84	Calibration
31	24 HPV-OHR CIN3	28 FIGO1A	0-100	14.3	0.84	Calibration
32	25 HPV-9V CIN3	1 Normal	0-100	5.7	0.84	Calibration
33	25 HPV-9V CIN3	28 FIGO1A	0-100	14.3	0.84	Calibration
34	26 HPV-18 CIN3	1 Normal	0-100	5.7	0.84	Calibration
35	26 HPV-18 CIN3	28 FIGO1A	0-100	14.3	0.84	Calibration
36	27 HPV-16 CIN3	1 Normal	0-100	5.7	0.84	Calibration
37	27 HPV-16 CIN3	28 FIGO1A	0-100	14.3	0.84	Calibration
38	28 Preclinical FIGO1A	29 Preclinical FIGO1B	0-100	4	1	Calibration
39	29 Preclinical FIGO1B	34 Clinical FIGO1B	0-100	2.2	1	Calibration
40	29 Preclinical FIGO1B	30 Preclinical FIGO2	0-100	2.2	1	Calibration
41	30 Preclinical FIGO2	35 Clinical FIGO2	0-100	1.7	1	Calibration
42	30 Preclinical FIGO2	31 Preclinical FIGO3	0-100	1.7	1	Calibration
43	31 Preclinical FIGO3	36 Clinical FIGO3	0-100	1.7	1	Calibration
44	31 Preclinical FIGO3	32 Preclinical FIGO4	0-100	1.7	1	Calibration
45	32 Preclinical FIGO4	37 Clinical FIGO4	0-100	0.7	1	Calibration

*All possible states and transitions are graphically presented in Figure 2.

HPV = human papillomavirus; OHR = other high risk types; 9V = HPV genotypes covered by the nonavalent vaccine, excluding HPV 16 and HPV 18; CIN = cervical intraepithelial neoplasia; FIGO = International Federation of Gynaecology and Obstetrics.

5.3 Screening part

For this analysis, three screening programmes are modelled: the cytology screening programme as it was applied in The Netherlands until 2016, the hrHPV-based programme that was implemented in The Netherlands in 2017 and the hrHPV-based programme with genotyping and increased referral threshold that was implemented in The Netherlands in July 2022.

In the modelled Dutch cytology programme, women aged 30–60 were screened five-yearly. Women with a High-grade Squamous Intraepithelial Lesion (HSIL) or worse were referred for colposcopy, while women with a lower grade positive cytology result were invited for a repeat test after six months using a co-test of cytology and hrHPV (Figure 5).

In the hrHPV screening programme, women are still invited five-yearly at the ages 30-60, however the screening interval is extended to ten years for women testing negative for hrHPV at the ages of 40 or 50 and there is an extra invitation at the age of 65 for women testing hrHPV+ at age 60. After a positive hrHPV test, the sample is analysed with cytology after which a positive cytology leads to referral to colposcopy, while after a negative cytology women are invited for a repeat cytology test after six months (Figure 6). Women who are uncomfortable with taking a test at their general practitioner can request a hrHPV-sampling kit, although after a positive self-sample, the women still need a smear at their GP to perform the cytology on.

In the hrHPV screening programme with genotyping and increased referral threshold, the referral threshold depends on the HPV type of the infection. Women with a HPV-16 or HPV-18 infection are still referred to a colposcopy if they have a positive cytology. Otherwise they are invited for a repeat cytology test after 12 months. If hrHPV+ women have a OHR or 9V infection, the referral threshold for a colposcopy is increased to Low-grade Squamous Intraepithelial Lesion (LSIL) or worse. Otherwise they are invited for a repeat cytology test after 12 months. At the repeat cytology, the threshold for a colposcopy is again a positive cytology (Figure 6).

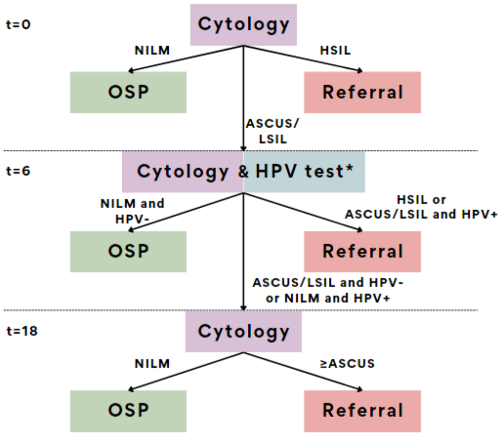

Figure 5 Triage in the cytology screening programme.

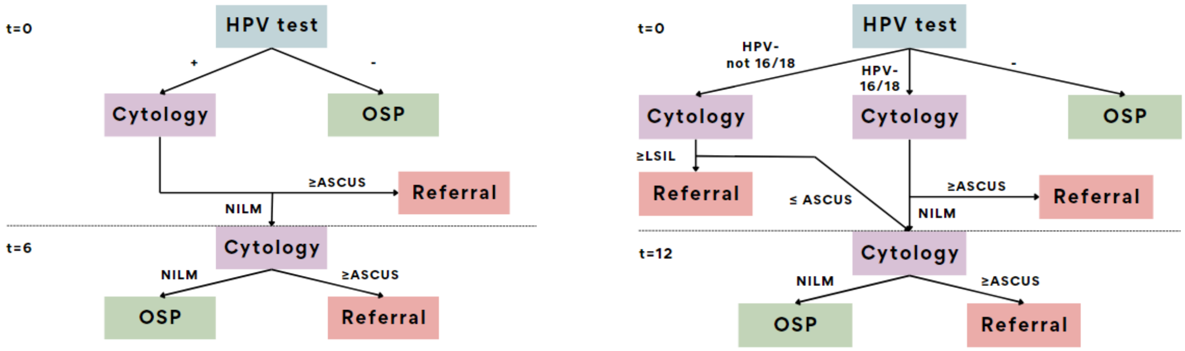

Figure 6 Triage in the hrHPV screening programme without (right) and with (left) genotyping and increased referral threshold.

t = Time in months since primary test; OSP = Organised screening programme; HPV = Human papillomavirus; NILM = Negative for intraepithelial lesion or malignancy; ASC-US = Atypical squamous cells of undetermined significance; LSIL = Low-grade squamous intraepithelial lesion; HSIL = High-grade squamous intraepithelial lesion.

Screening behavior

The age-specific probability to attend primary screening is based on observations in the cytology screening programme and both of the hrHPV-based screening programmes as well as the adherence to repeat testing or colposcopy in the triage. The probability to attend a primary cytology test was defined

as the proportion of women eligible for screening in 2015 that attended before April 1 2016. The probability to attend a primary hrHPV-test was defined as the proportion of women eligible for screening in 2017 that attended before August 2018 either at their GP or by returning a self-sampling kit. The three months of longer follow-up for the hrHPV-based programme were added because the hrHPV-programme was starting up in 2017 and some delays in attendance might have occurred because of the implementation phase. We did not model any opportunistic screening activities outside the age ranges of the screening programmes. The adherence to repeat testing was defined as the proportion of women referred to repeat testing having a registered surveillance test within 6 months after the recommended date of the repeat test. The adherence to colposcopy was defined as the proportion of women referred to colposcopy having a registered cytology or histology result within 15 months after the referral date. The reason for this long follow-up is that it is standard practice in The Netherlands to not perform a biopsy at women having a low grade cytology result and a clear colposcopy, but to invite them for a repeat cytology after 12 months instead.

Test characteristics

The test characteristics in MISCAN-Cervix are presented in Table 3. The probabilities of a cytology test being positive are calibrated on CIN detection rates and interval cancers in the Dutch screening program. The calibration process is explained in more detail in the calibration section. Test characteristics for the hrHPV-test are based on literature. The sensitivity of the hrHPV test for hrHPV-positive women with a ≥CIN2 lesion was found to be 94% in the POBASCAM study.[18] The probability of a positive hrHPV-test in hrHPV-positive women with no CIN and hrHPV positive women with a CIN1 is based on a study of Rebolj and colleagues using data from the Danish Horizon study.[19] The study presented concordance between the Hybrid Capture 2, cobas, CLART and Aptima hrHPV tests. We assumed that any prevalent hrHPV infection would be picked up by at least one of the four assays and defined the probability of a positive test result in the model as the proportion of those total hrHPV infections that tested positive on the cobas hrHPV test (since this test was used in the Dutch program in 2017-2023).

Table 3 Test characteristics of the cytology test and the hrHPV-test by disease status.

**Test result and disease status**	**Probability of a positive test result (age<50)**	**Probability of a positive test result (age≥50)**
*Positive hrHPV-test**, in case of no prevalent hrHPV infection*	0%	0%
*Positive hrHPV-test**, in case of ≥1 prevalent hrHPV infection*		
- No CIN present	69%	69%
- CIN1	72%	72%
- CIN2	94%	94%

- CIN3<br>- Cervical cancer	94%<br>94%	94%<br>94%
*Cytology ≥ASC-US in case of no prevalent hrHPV infection**<br>- No CIN present<br>- CIN1<br>- CIN2<br>- CIN3<br>- Cervical cancer	 0.60%***<br>36.24%***<br>37.13%***<br>75.40%<br>85.09%	 0.60%***<br>15.67%***<br>24.08%***<br>65.21%<br>85.09%
*Cytology ≥ASC-US in case of ≥1 prevalent hrHPV infection**<br>- No CIN present<br>- CIN1<br>- CIN2<br>- CIN3<br>- Cervical cancer	 17.08%***<br>36.24%***<br>37.13%***<br>75.40%<br>85.09%	 8.54%***<br>15.67%***<br>24.08%***<br>65.21%<br>85.09%
*Cytology ≥ HSIL in case of no prevalent hrHPV infection**<br>- No CIN present<br>- CIN1<br>- CIN2<br>- CIN3<br>- Cervical cancer	 0.04%<br>2.62%<br>10.72%<br>51.61%<br>64.73%	 0.04%<br>2.62%<br>10.72%<br>51.61%<br>64.73%
*Cytology ≥ HSIL in case of ≥1 prevalent hrHPV infection**<br>- No CIN present<br>- CIN1<br>- CIN2<br>- CIN3<br>- Cervical cancer	 0.00%<br>2.62%<br>10.72%<br>51.61%<br>64.73%	 0.00%<br>1.13%<br>6.95%<br>44.63%<br>64.73%
**Test result and disease status**	**Probability of a positive test result (age<50)**	**Probability of a positive test result (age≥50)**
*Positive hrHPV-test**, in case of no prevalent hrHPV infection*	0%	0%
*Positive hrHPV-test**, in case of ≥1 prevalent hrHPV infection*<br>- No CIN present	 69%	 69%

- CIN1	72%	72%
- CIN2	94%	94%
- CIN3	94%	94%
- Cervical cancer	94%	94%
*Cytology ≥ASC-US in case of no prevalent hrHPV infection**		
- No CIN present	0.60%***	0.60%***
- CIN1	36.24%***	15.67%***
- CIN2	37.13%***	24.08%***
- CIN3	75.40%	65.21%
- Cervical cancer	85.09%	85.09%
*Cytology ≥ASC-US in case of ≥1 prevalent hrHPV infection**		
- No CIN present	17.08%***	8.54%***
- CIN1	36.24%***	15.67%***
- CIN2	37.13%***	24.08%***
- CIN3	75.40%	65.21%
- Cervical cancer	85.09%	85.09%
*Cytology ≥ HSIL in case of no prevalent hrHPV infection**		
- No CIN present	0.04%	0.04%
- CIN1	2.62%	2.62%
- CIN2	10.72%	10.72%
- CIN3	51.61%	51.61%
- Cervical cancer	64.73%	64.73%
*Cytology ≥ HSIL in case of ≥1 prevalent hrHPV infection**		
- No CIN present	0.00%	0.00%
- CIN1	2.62%	1.13%
- CIN2	10.72%	6.95%
- CIN3	51.61%	44.63%
- Cervical cancer	64.73%	64.73%

*Probability to test positive the first time a woman with this lesion present attends screening. 12% of the CIN lesions will be missed systematically over time (calibrated). 24% of the CIN lesions will be missed systematically over time for women aged 50 or older (calibrated).

** The same test characteristics are assumed for GP smears as for self-sampling kits.

*** The sensitivity of the cytology test is lower for low-grade lesions for women aged 50 or older. For CIN0 and CIN1 50% of the original sensitivity and for CIN2 75% of the original sensitivity.

hrHPV = high-risk human papillomavirus; CIN = cervical intraepithelial neoplasia; ASC-US = Atypical squamous cells of undetermined significance; LSIL = Low-grade squamous intraepithelial lesion; HSIL = High-grade squamous intraepithelial lesion.

6. Model (re-)calibration

The values of some model parameters in the parameter overview could either be based on observed data or on available literature. However, some other model parameters could not be derived from observational data (e.g. the age-specific background risk for acquiring an hrHPV-infection). Therefore, these parameter values needed to be calibrated. For this study, not all calibrated parameters are (re-)calibrated. See the previous model description for information on the calibrated parameters that are not mentioned.[1]

In this calibration of MISCAN-Cervix, a population is simulated for which high quality observational data is available including the screening behaviour of that population. In this case we used the Dutch female population (born in 1904-2022) in 2004-2022 and simulated their screening behaviour in the cytology programme and the hrHPV programme.

The parameters that could not be derived from observational data or literature are estimated based on expert opinion or studies on similar parameters. The model runs with this set of parameter values after which the outputs of the model are compared with the observed data of that population on:

Table 4 Calibration targets with corresponding target years and their sources.

Target	Target years	Source
**Cervical cancer stage distribution by age**	2004-2013 & 2017-2021	Netherlands Cancer Registry (NCR) & the National Institute for Public Health and the Environment (RIVM)
**Cervical cancer incidence by age**	2004-2013	Netherlands Cancer Registry (NCR)
**HPV prevalence by age**	2017-2021	the National Institute for Public Health and the Environment (RIVM)
**Longitudinal HPV prevalence**	2022	Bevolkingsonderzoek Nederland (ScreenIT)
**HPV genotype distribution**	2017-2021	the National Institute for Public Health and the Environment (RIVM)

**Histology result by HPV type**	2017-2021	the National Institute for Public Health and the Environment (RIVM)
**Histology result by lesion grade and rank**	2004-2013	Netherlands Cancer Registry (NCR)

Based on this comparison, the model inputs are adjusted using a Genetic Algorithm. With these new inputs the model runs again where the calibration cycle starts again. This cycle is repeated until the outputs of the model reflect the observed data well.

6.1 Method

The model needed to be recalibrated because the first results of the second hrHPV screening round have recently become known. This showed that 50% of the women who were invited at age 65, after a positive test without referral at age 60, had a positive test again in the second round. In the MISCAN-Cervix model, it was modeled that approximately 10% of those women received a positive test result at age 65.

To ensure the model fits (better) on this data, we multiplied the probability of systematically missing a CIN with the cytology test with a factor for women aged 50 years or older. Moreover, most importantly, we added that the duration of an hrHPV infection increases with age, as explained in 5.2. To ensure that the model still fit the HPV prevalence and cervical cancer incidence data, we also had to recalibrate the age factors and the regression probabilities of hrHPV infections. This is due to the fact that if the durations of hrHPV infections are longer, there are less infections necessary to fit the HPV prevalence and in turn less regression to fit the cervical cancer incidence. Since the duration is dependent on age, but not on hrHPV genotype, we calibrated a factor on the age factors and regression probabilities based on age categories.

Additionally, we noticed a lack of heterogeneity in the hrHPV genotype distribution. The individual hazard is now taken from a Gamma distribution with a mean of 1 for the general population and 2.6 for the 'never attenders'. The variance is calibrated and equal for both groups.

The calibrated parameters related to the development of hrHPV infections are as follows:

- hrHPV duration mean for ages 0-29, 30-39, 40-49, 50-100
- hrHPV duration shape for ages 0-29, 30-39, 40-49, 50-100
- Factor on the age factors for an hrHPV infection for ages 0-15, 16-20, 21-25, 26-30, 31-35, 36-50, 51-65

- Factor on the regression probabilities of an hrHPV infection for ages 0-15, 16-25, 26-35, 36-50, 51-75
- Variance of the Gamma distribution for the individual hazard

The test characteristic calibrated is:

- Probability that the cytology test is systematically negative for women aged 50 and older

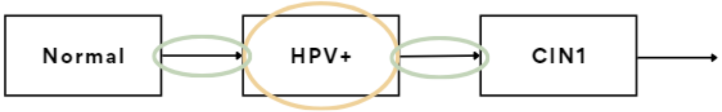

Figure 7 Calibrated durations (yellow) and probabilities (green) during the calibration process.

HPV = human papillomavirus; CIN = cervical intraepithelial neoplasia

7. Calibration results

The Dutch MISCAN-Cervix model was calibrated to data with sources as given in Table . Furthermore, the model predictions on age-specific mortality fitted well with the observed age-specific mortality rates in the Netherlands from 2004-2013 from the NCR, which were not used to fit on during the calibration, showing the validity of the model.

Below we present the calibration results with the observed data in blue with corresponding confidence interval and the simulated data in black.

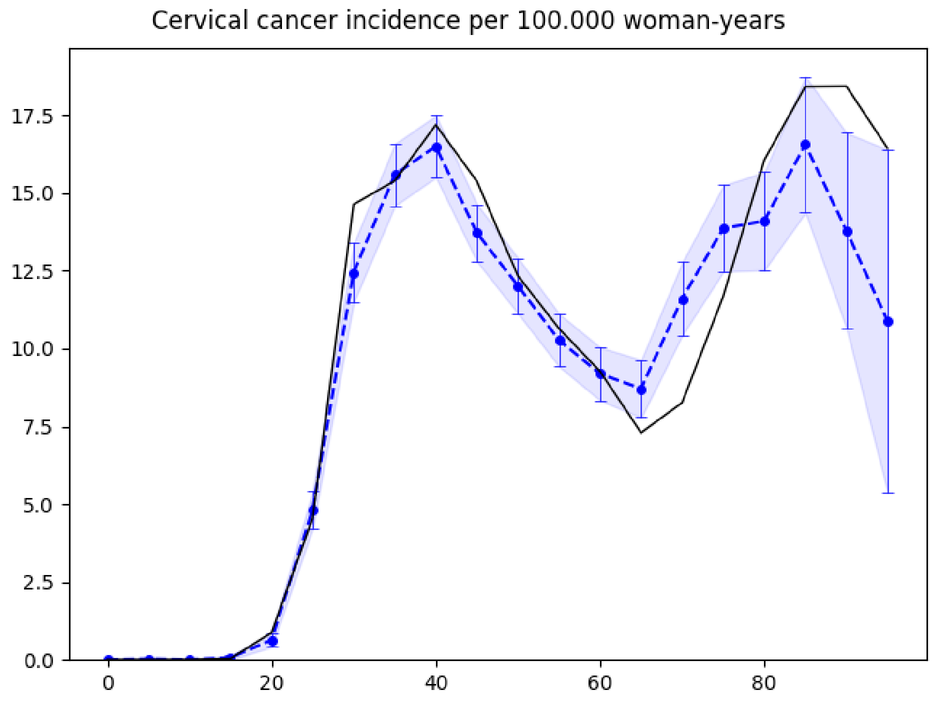

Figure 8 Model fit on age-specific cervical cancer incidence rates.

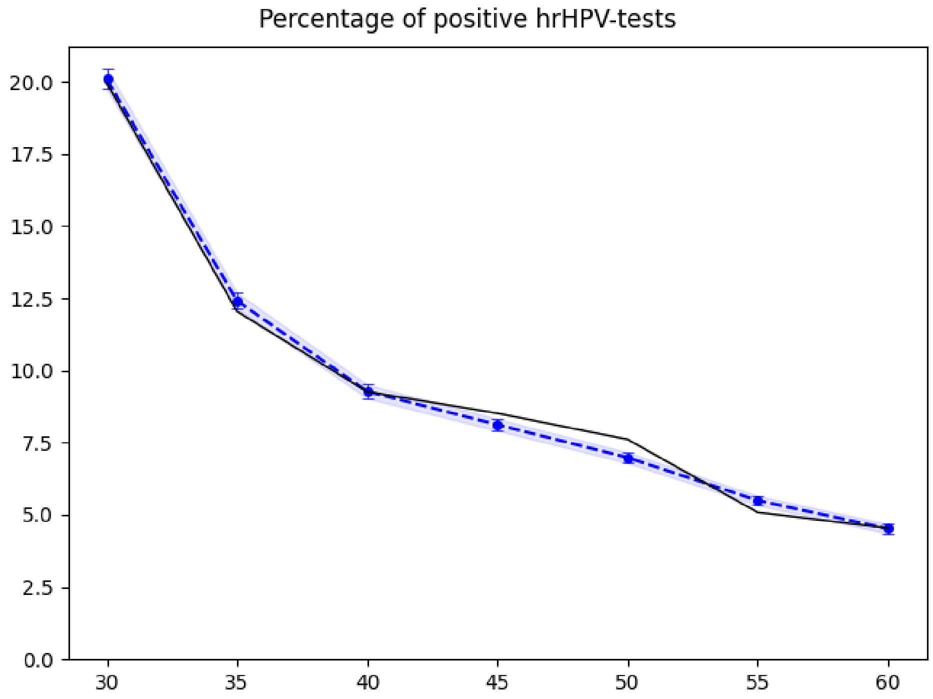

Figure 9 Model fit on age-specific hrHPV test-positivity rates.

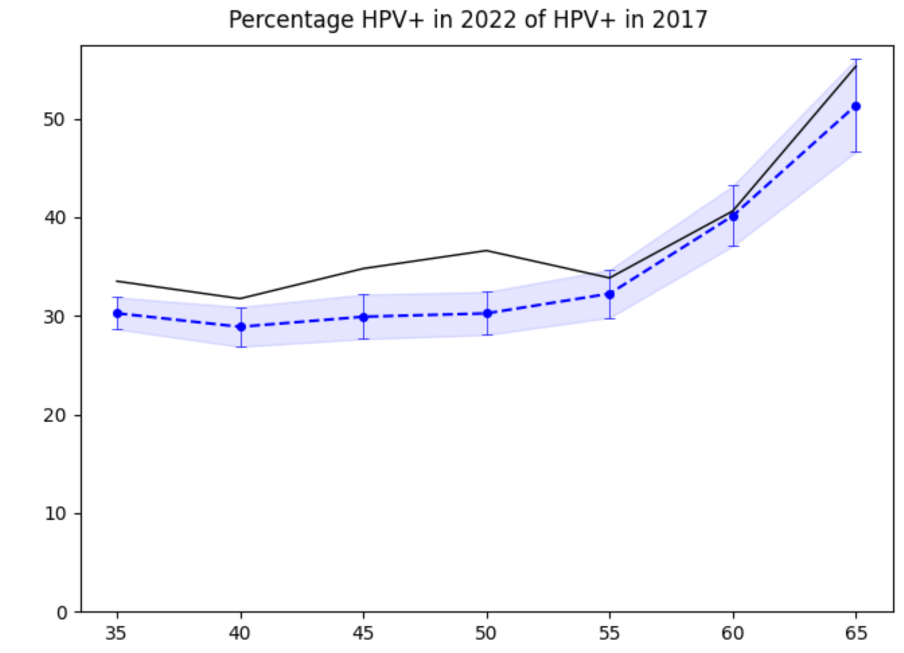

Figure 10 Model fit on age-specific hrHPV test-positivity rates for women who also tested positive 5 years before.

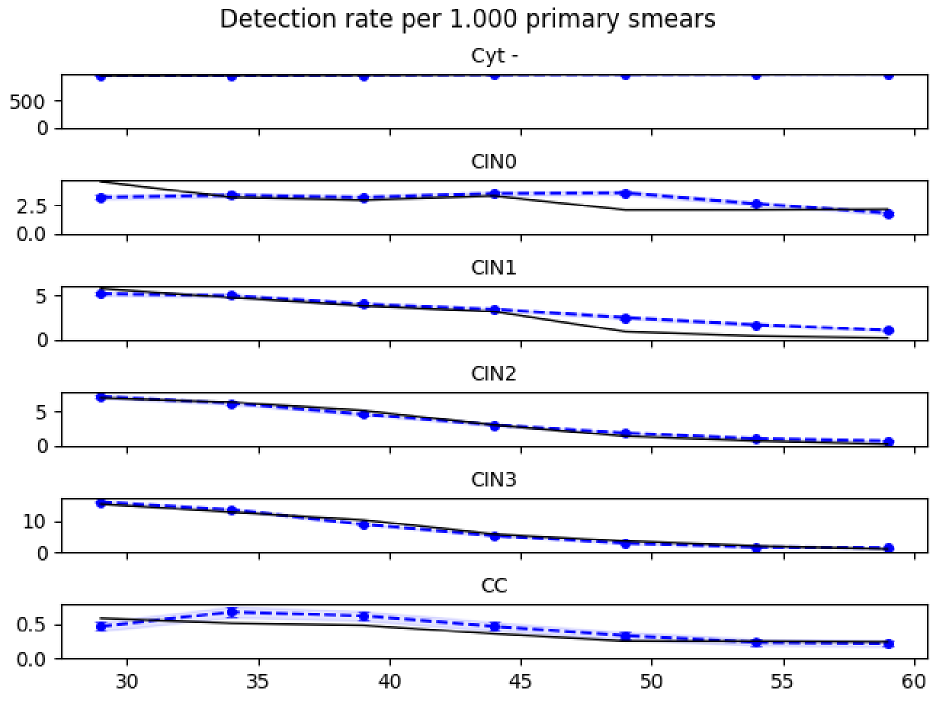

Figure 11 Model fit on age-specific detection rates.

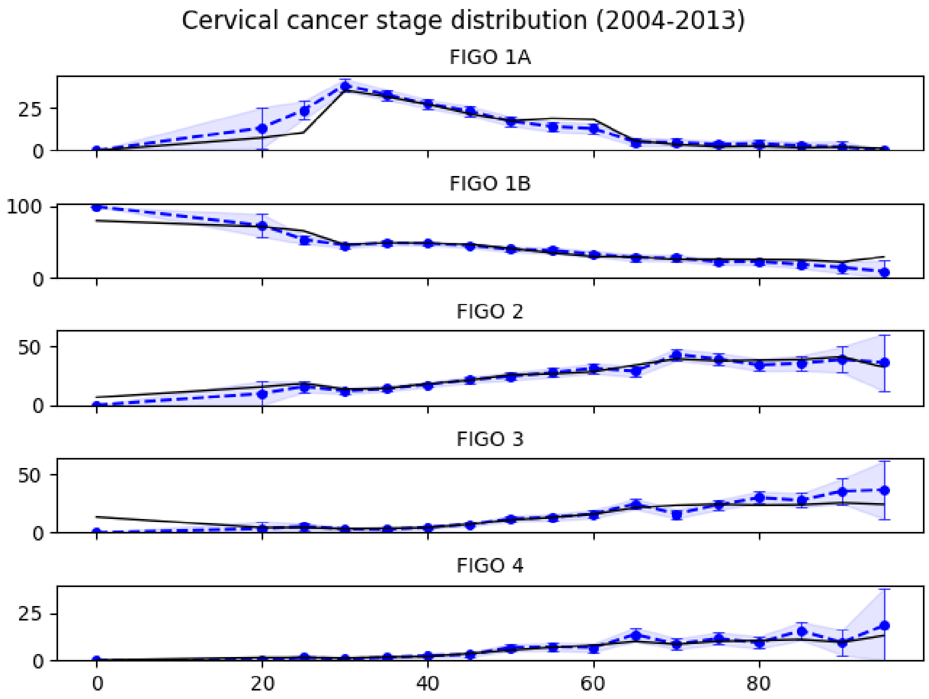

Figure 12 Model fit on cervical cancer stage distribution by age (2004-2013).

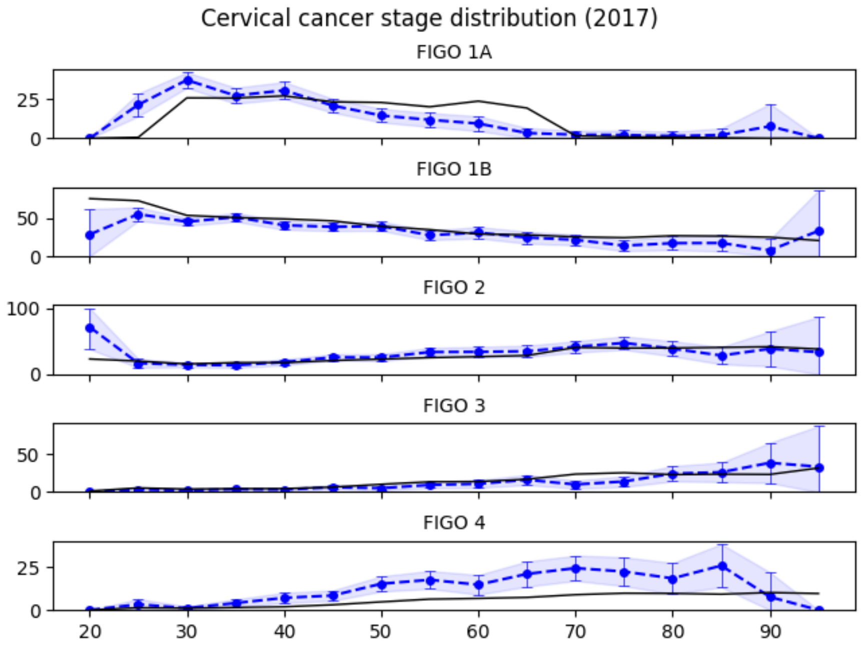

Figure 13 Model fit on cervical cancer stage distribution by age (2017).

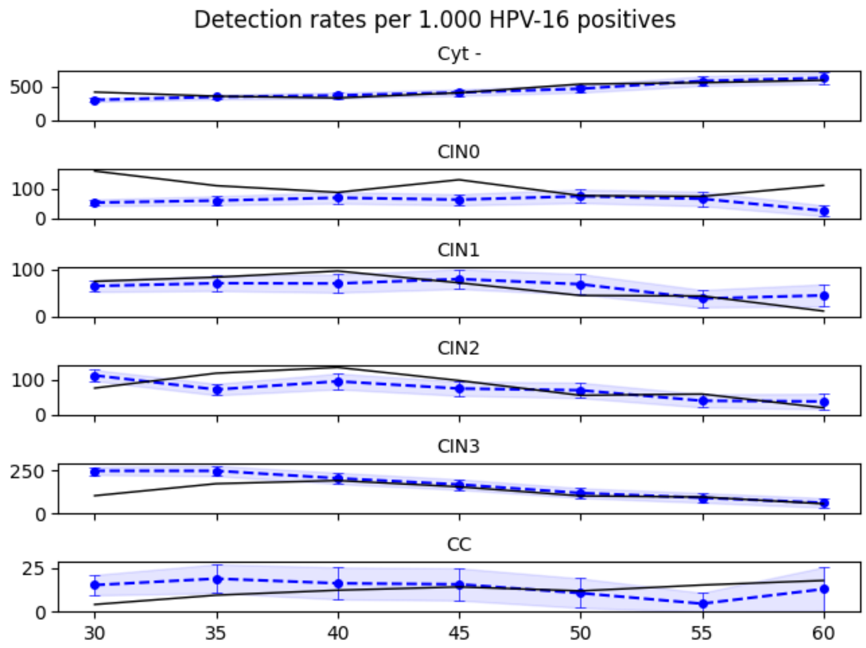

Figure 14 Model fit on age-specific detection rates for women who tested positive for HPV-16.

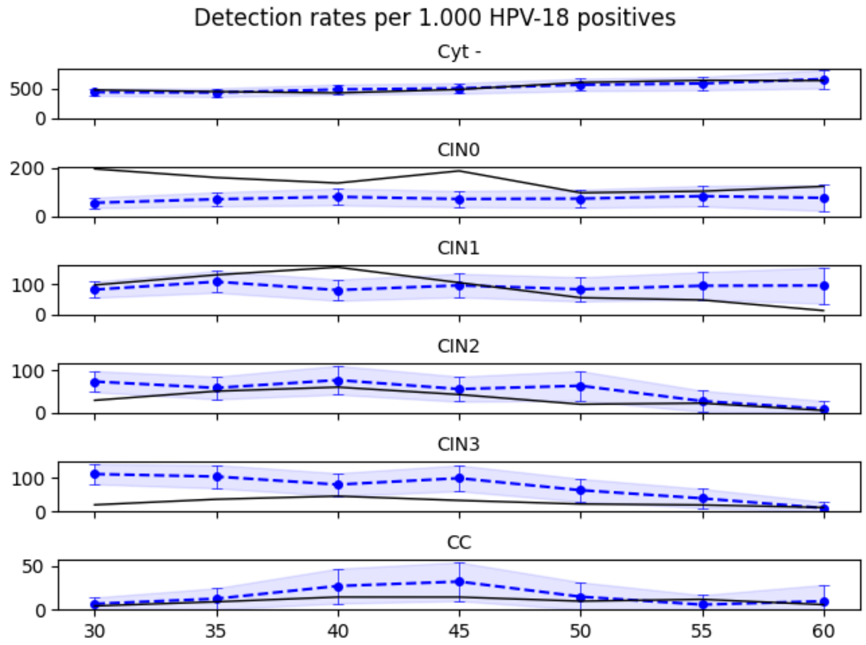

Figure 15 Model fit on age-specific detection rates for women who tested positive for HPV-18.

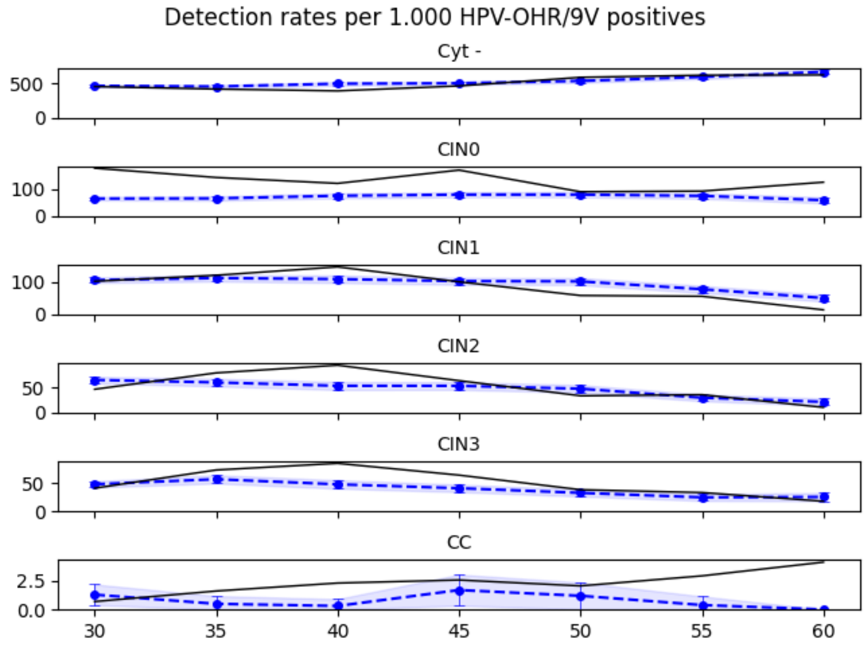

Figure 16 Model fit on age-specific detection rates for women who tested positive for HPV-OHR/9V.

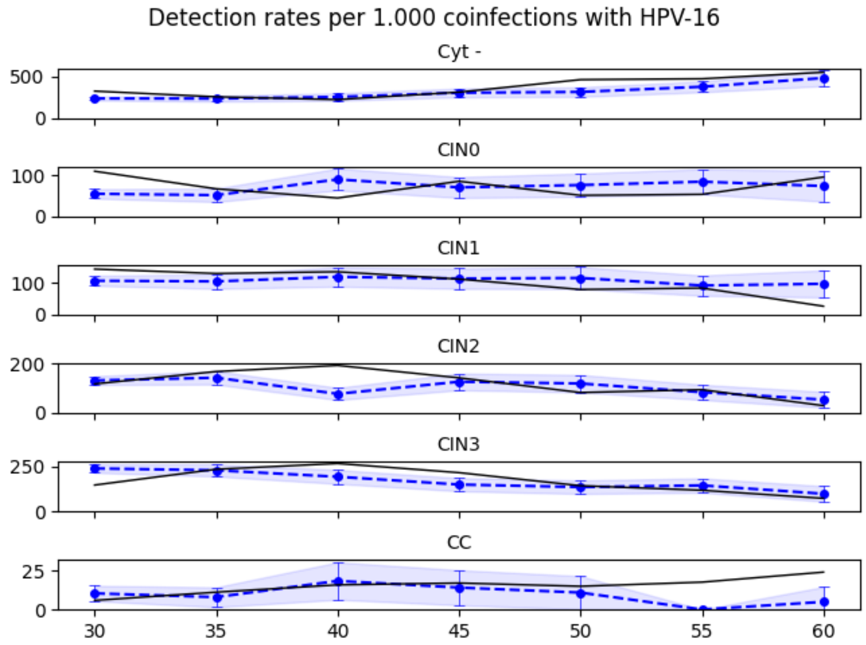

Figure 17Model fit on age-specific detection rates for women who tested positive for a coinfection with HPV-16.

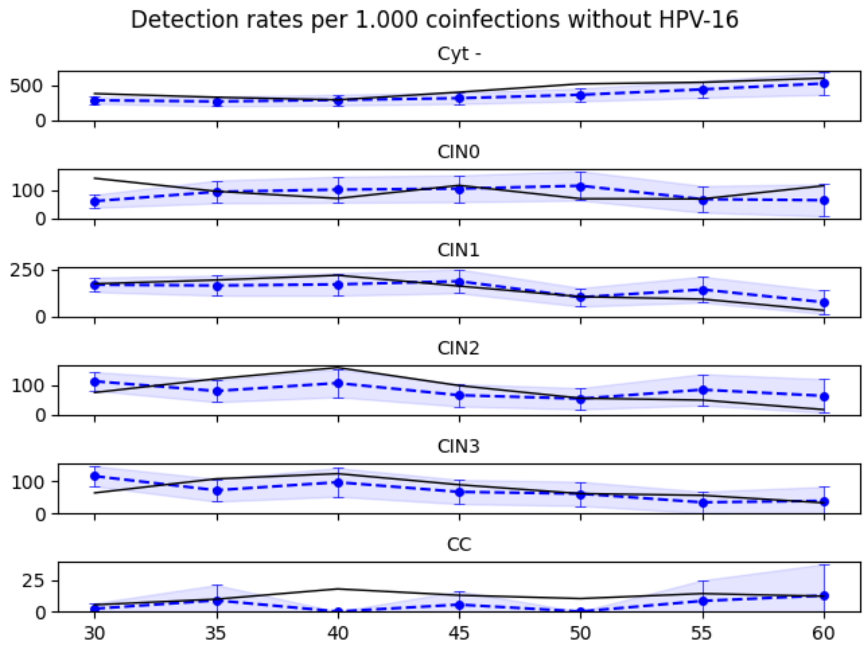

Figure 18 Model fit on age-specific detection rates for women who tested positive for coinfections without HPV-16.

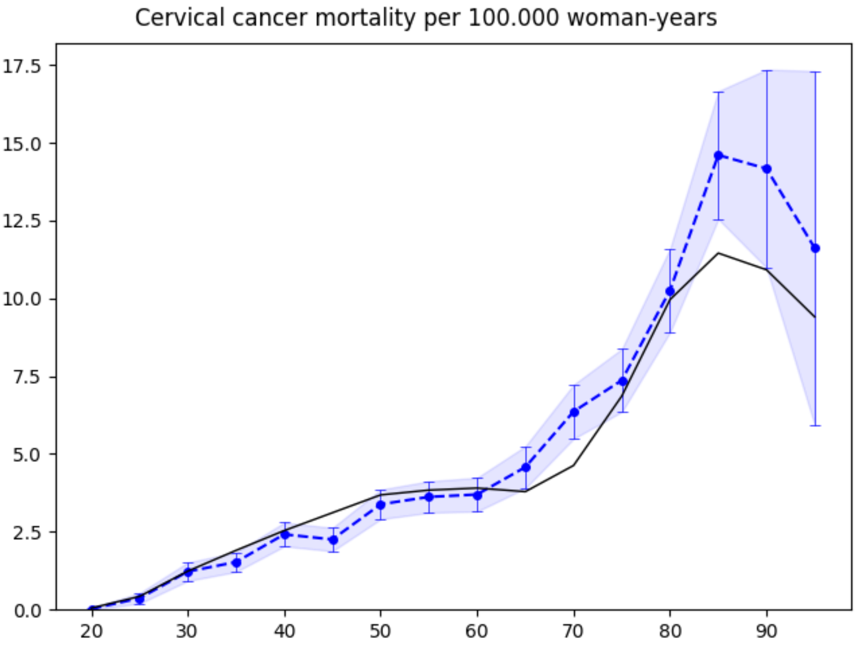

Figure 19 Model fit on age-specific mortality rates.

2.2

References

1. Jansen E, Naber SK, Aitken CA, de Koning HJ, van Ballegooijen M, de Kok I. Cost-effectiveness of HPV-based cervical screening based on first year results in the Netherlands: a modelling study. BJOG. 2021 Feb;128(3):573-82.
2. Andrae B, Kemetli L, Sparen P, Silfverdal L, Strander B, Ryd W, et al. Screening-preventable cervical cancer risks: evidence from a nationwide audit in Sweden. J Natl Cancer Inst. 2008 May 7;100(9):622-9.
3. Landy R, Pesola F, Castanon A, Sasieni P. Impact of cervical screening on cervical cancer mortality: estimation using stage-specific results from a nested case-control study. Br J Cancer. 2016 Oct 25;115(9):1140-6.
4. Lonnberg S, Nieminen P, Luostarinen T, Anttila A. Mortality audit of the Finnish cervical cancer screening program. Int J Cancer. 2013 May 1;132(9):2134-40.
5. Habbema JD, van Oortmarssen GJ, Lubbe JT, van der Maas PJ. The MISCAN simulation program for the evaluation of screening for disease. Comput Methods Programs Biomed. 1985 May;20(1):79-93.
6. Draisma G, Etzioni R, Tsodikov A, Mariotto A, Wever E, Gulati R, et al. Lead time and overdiagnosis in prostate-specific antigen screening: importance of methods and context. J Natl Cancer Inst. 2009 Mar 18;101(6):374-83.
7. Loeve F, Boer R, van Oortmarssen GJ, van Ballegooijen M, Habbema JD. The MISCAN-COLON simulation model for the evaluation of colorectal cancer screening. Comput Biomed Res. 1999 Feb;32(1):13-33.
8. Ten Haaf K, de Koning HJ. Should Never-Smokers at Increased Risk for Lung Cancer Be Screened? J Thorac Oncol. 2015 Sep;10(9):1285-91.
9. van den Akker-van Marle ME, van Ballegooijen M, van Oortmarssen GJ, Boer R, Habbema JD. Cost-effectiveness of cervical cancer screening: comparison of screening policies. J Natl Cancer Inst. 2002 Feb 6;94(3):193-204.
10. van der Maas PJ, de Koning HJ, van Ineveld BM, van Oortmarssen GJ, Habbema JD, Lubbe KT, et al. The cost-effectiveness of breast cancer screening. Int J Cancer. 1989 Jun 15;43(6):1055-60.
11. Walboomers JM, Jacobs MV, Manos MM, Bosch FX, Kummer JA, Shah KV, et al. Human papillomavirus is a necessary cause of invasive cervical cancer worldwide. J Pathol. 1999 Sep;189(1):12-9.
12. Quint W, Jenkins D, Molijn A, Struijk L, van de Sandt M, Doorbar J, et al. One virus, one lesion--individual components of CIN lesions contain a specific HPV type. J Pathol. 2012 May;227(1):62-71.
13. Levensverwachting; geslacht, leeftijd (per jaar en periode van vijf jaren). 2023 [cited 2023; Available from: https://opendata.cbs.nl/statline/#/CBS/nl/dataset/37360ned/table?fromstatweb
14. Operaties in het ziekenhuis; soort opname, leeftijd en geslacht, 1995-2010. 2014 [cited 2019; Available from: https://opendata.cbs.nl/statline/#/CBS/nl/dataset/80386ned/table?ts=1528353487106

15. Coupe VM, Berkhof J, Bulkmans NW, Snijders PJ, Meijer CJ. Age-dependent prevalence of 14 high-risk HPV types in the Netherlands: implications for prophylactic vaccination and screening. Br J Cancer. 2008 Feb 12;98(3):646-51.
16. Moscicki AB, Shiboski S, Hills NK, Powell KJ, Jay N, Hanson EN, et al. Regression of low-grade squamous intra-epithelial lesions in young women. Lancet. 2004 Nov 6-12;364(9446):1678-83.
17. Moscicki AB, Ma Y, Wibbelsman C, Darragh TM, Powers A, Farhat S, et al. Rate of and risks for regression of cervical intraepithelial neoplasia 2 in adolescents and young women. Obstet Gynecol. 2010 Dec;116(6):1373-80.
18. Bulk S, Bulkmans NW, Berkhof J, Rozendaal L, Boeke AJ, Verheijen RH, et al. Risk of high-grade cervical intra-epithelial neoplasia based on cytology and high-risk HPV testing at baseline and at 6-months. Int J Cancer. 2007 Jul 15;121(2):361-7.
19. Rebolj M, Bonde J, Preisler S, Ejegod D, Rygaard C, Lynge E. Differential Detection of Human Papillomavirus Genotypes and Cervical Intraepithelial Neoplasia by Four Commercial Assays. J Clin Microbiol. 2016 Nov;54(11):2669-75.

Appendix B: Calibration settings and results

For the calibration of cohort effects we used the genetic algorithm with the following settings:

- Sample size for each run = 1e7
- Starting population = 40
- Generation size = 16
- Size of hall of fame (list of best fits) = 8
- Seed = 1234
- Number of generations = 40
- Tournament size = 2
- Crossover probability = 0.8
- Mutation probability = 0.6
- Starting factors (cohorts 1978-1982, 1983-1987, 1988-1992, 1993+) = [1.2, 1.2, 1.2, 1.2]
- Lower limit for factors: [1, 1, 1, 1]
- Upper limit for factors: [2, 2, 2, 2]

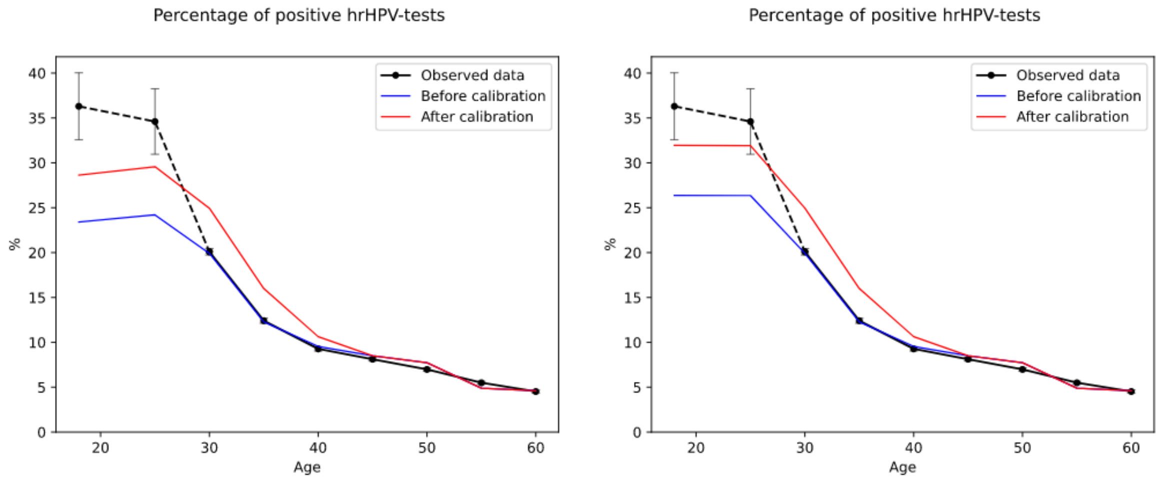

Figure 1. *Percentage of positive hrHPV-tests in the Netherlands, observed data compared with the model outcomes before and after calibration of the cohort effect. The model outcomes in the left figure represent the Dutch situation with a partly-vaccinated population, while those in the right figure represent a situation without vaccination. The observed data above age 30 is based on Dutch screening programme data from 2017-2018 and the observed data below age 30 is based on hrHPV prevalence in Finland.*[1]

References:

1. Leinonen MK, Anttila A, Malila N, Dillner J, Forslund O, Nieminen P. Type- and age-specific distribution of human papillomavirus in women attending cervical cancer screening in Finland. Br J Cancer 2013;109: 2941-50.

Appendix C: Assumptions for costs, QALYs lost and vaccination rate

Table 1: Assumptions for costs and QALYs lost

Assumptions for costs are based on the actual costs in the Dutch healthcare system. Assumptions for disutilities are based on De Kok et al. (2018).[1]

	**Total cost (euros)***	**Disutility per year**	**Duration disutility in months**
**SCREENING**			
Primary hrHPV test	69	0	0
Primary hrHPV self-sampling	52	0	0
Reflex cytology after hrHPV-test	31	0	0
Repeat cytology after hrHPV self-sampling	63	0.03	1
Repeat cytology after 12 months	64	0.03	12
**DIAGNOSE AND TREATMENT**			
No CIN	380	0.03	1
CIN1	1185	0.03	1
CIN2	1755	0.03	1
CIN3	2055	0.03	1
FIGO1A	6731	0.08	12
FIGO1B	15961	0.08	12
FIGO2+ clinically detected	14692	0.14	12
FIGO2+ screen detected	15732	0.14	12
Cancer survivor	0	0.03	120
Palliative care	35744	0.5	12

hrHPV = high-risk human papillomavirus; CIN = cervical intraepithelial neoplasia; FIGO = International Federation of Gynecology and Obstetrics

References:

1. de Kok I, Korfage IJ, van den Hout WB, Helmerhorst TJM, Habbema JDF, Essink-Bot ML, van Ballegooijen M. Quality of life assumptions determine which cervical cancer screening strategies are cost-effective. Int J Cancer 2018;142: 2383-93.

Table 2: Vaccination rates (fully vaccinated, 2 or 3 doses)

	**Calendar year**										
**Birth cohort**	2008	2009	2010	2011	2012	2013	2014	2015	2016	2017	2018
1993	0	0	0.49	0	0	0	0	0	0	0	0
1994	0	0	0.525	0	0	0	0	0	0	0	0
1995	0	0	0.538	0	0	0	0	0	0	0	0
1996	0	0	0.542	0	0	0	0	0	0	0	0
1997	0	0	0	0.56	0	0	0	0	0	0	0
1998	0	0	0	0	0.581	0	0	0	0	0	0
1999	0	0	0	0	0	0.589	0	0	0	0	0
2000	0	0	0	0	0	0	0.61	0	0	0	0
2001	0	0	0	0	0	0	0	0.61	0	0	0
2002	0	0	0	0	0	0	0	0	0.534	0	0
2003	0	0	0	0	0	0	0	0	0	0.455	0
>=2004	0	0	0	0	0	0	0	0	0	0	0.455*

* Women are vaccinated at age 13.

Appendix D: Results for sensitivity analysis without cohort effect

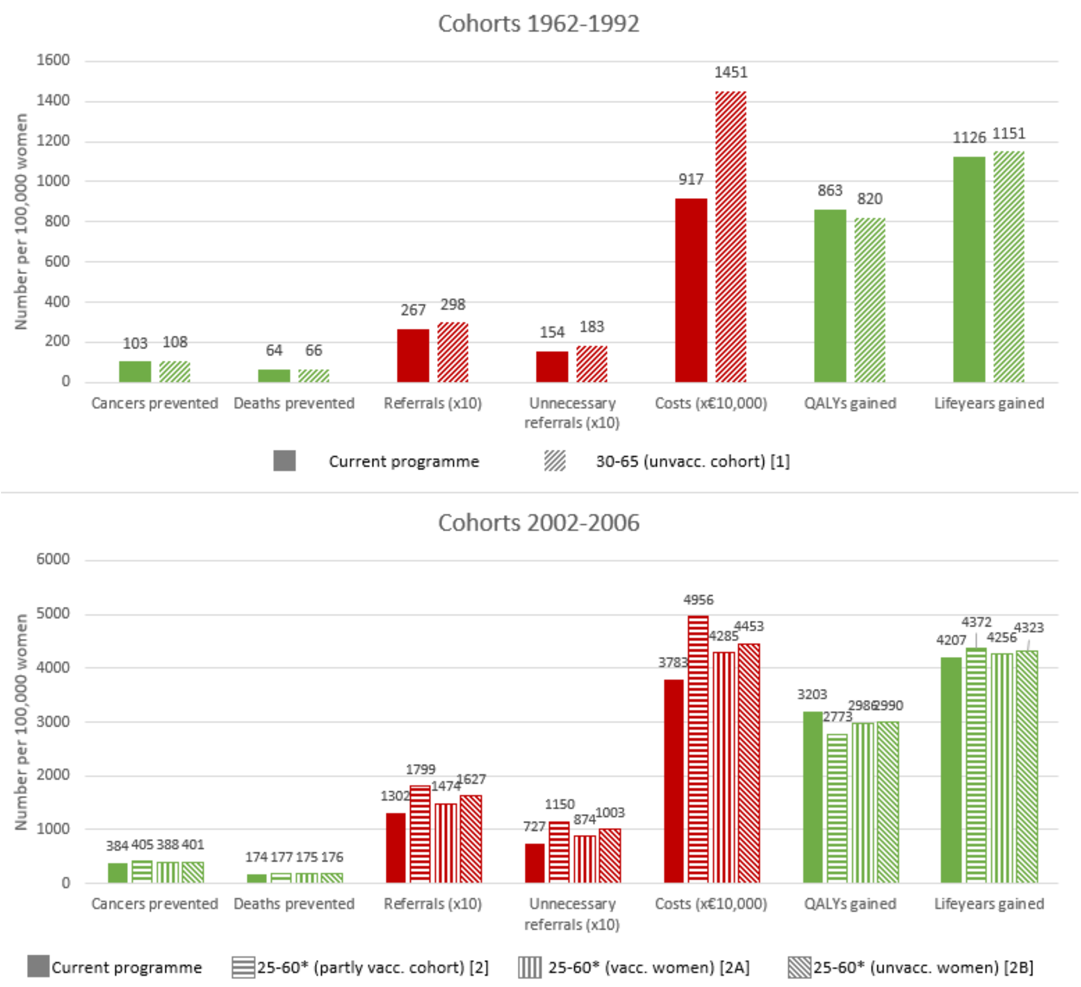

Figure 1 Harms and benefits for unvaccinated cohorts (top panel) and partly vaccinated cohorts (bottom panel) for sensitivity analysis without cohort effect. * Women are additionally screened at age 65 if they tested HPV-positive at age 60 and were not referred to a gynecologist.

Table 1 Program efficiency and cost-effectiveness comparison between strategies and current program for sensitivity analysis without cohort effect. HPV: human papillomavirus, PPV HPV: positive predictive value of the primary HPV test, NNS CIN2/3+: Number needed to screen to find one CIN2/3+, NNR CIN2/3+: Number needed to refer to find one CIN2/3+, CER: cost-effectiveness ratio.

		**CIN2+ efficiency**			**CIN3+ efficiency**			**Cost-effectiveness**
**Nr.**	**Strategy**	**PPV HPV+[a]**	**NNS[b]**	**NNR[c]**	**PPV HPV+[a]**	**NNS[b]**	**NNR[c]**	**ICER compared to current program**
1962-1992	Current program	10.2%	118.0	2.4	5.3%	226.3	4.5	
**1**	30-65 (unvaccinated cohorts)	8.5%	180.7	2.6	4.4%	346.7	5.0	€368,709/LYG
2002-2006	Current program	12.9%	76.9	2.3	6.7%	148.9	4.4	
**2**	25-60 (mixed cohorts)	9.9%	82.1	2.8	4.8%	170.6	5.8	€180,745/LYG
**2A**	25-60 (vaccinated women)	11.3%	80.8	2.5	5.7%	159.9	4.9	€271,481/LYG
**2B**	25-60 (unvaccinated women)	11.0%	78.6	2.6	5.4%	160.2	5.3	€144,680/LYG

[a] Positive predictive value of the primary Human Papillomavirus (HPV) test. Calculated as the number of CIN2+/3+ lesions found after a positive HPV screening result divided by the total number of positive HPV tests.

[b] Number needed to screen to find one CIN2+/3+ lesion. Calculated as the number of HPV screening tests performed divided by the number of CIN2+/3+ lesions found in the screening program.

[c] Number needed to refer to find one CIN2+/3+ lesion. Calculated as the number of referrals to the gynecologist divided by the number of CIN2+/3+ lesions found in the screening program.
